# DrugRep-HeSiaGraph: when heterogenous siamese neural network meets knowledge graphs for drug repurposing

**DOI:** 10.1186/s12859-023-05479-7

**Published:** 2023-10-03

**Authors:** Zahra Ghorbanali, Fatemeh Zare-Mirakabad, Najmeh Salehi, Mohammad Akbari, Ali Masoudi-Nejad

**Affiliations:** 1 Computational Biology Research Center (CBRC), Department of Mathematics and Computer Science, Amirkabir University of Technology, Tehran, Iran; 2 School of Biological Science, Institute for Research in Fundamental Sciences (IPM), Tehran, Iran; 3 Laboratory of Systems Biology and Bioinformatics (LBB), Institute of Biochemistry and Biophysics, University of Tehran, Tehran, Iran

**Keywords:** Drug repositioning, Deep learning, Graph embedding, Heterogenous network, COVID-19, Dipeptidyl peptidase 4

## Abstract

**Background:**

Drug repurposing is an approach that holds promise for identifying new therapeutic uses for existing drugs. Recently, knowledge graphs have emerged as significant tools for addressing the challenges of drug repurposing. However, there are still major issues with constructing and embedding knowledge graphs.

**Results:**

This study proposes a two-step method called DrugRep-HeSiaGraph to address these challenges. The method integrates the drug-disease knowledge graph with the application of a heterogeneous siamese neural network. In the first step, a drug-disease knowledge graph named DDKG-V1 is constructed by defining new relationship types, and then numerical vector representations for the nodes are created using the distributional learning method. In the second step, a heterogeneous siamese neural network called HeSiaNet is applied to enrich the embedding of drugs and diseases by bringing them closer in a new unified latent space. Then, it predicts potential drug candidates for diseases. DrugRep-HeSiaGraph achieves impressive performance metrics, including an AUC-ROC of 91.16%, an AUC-PR of 90.32%, an accuracy of 84.63%, a BS of 0.119, and an MCC of 69.31%.

**Conclusion:**

We demonstrate the effectiveness of the proposed method in identifying potential drugs for COVID-19 as a case study. In addition, this study shows the role of dipeptidyl peptidase 4 (DPP-4) as a potential receptor for SARS-CoV-2 and the effectiveness of DPP-4 inhibitors in facing COVID-19. This highlights the practical application of the model in addressing real-world challenges in the field of drug repurposing. The code and data for DrugRep-HeSiaGraph are publicly available at https://github.com/CBRC-lab/DrugRep-HeSiaGraph.

**Supplementary Information:**

The online version contains supplementary material available at 10.1186/s12859-023-05479-7.

## Background

The lengthy process and exorbitant cost of drug discovery have led researchers to explore the possibility of drug repositioning or drug repurposing (DR), i.e., investigating the potential therapeutic uses of existing medications beyond their original intended purpose. DR has gained much attention from researchers due to its potential to expedite the treatment process. Retrospective studies have highlighted several examples of drugs that have been successfully repurposed for new therapeutic uses. Thalidomide, originally developed as a sedative, has been repurposed for the treatment of leprosy, multiple myeloma, and other types of cancer [1]. Metformin was prescribed as an anti-diabetic medication and has been repurposed for the treatment of cancer and polycystic ovary syndrome [2]. These examples demonstrate the potential of DR to uncover new therapeutic uses for existing medications, which can lead to improve patient outcomes and reduce healthcare costs.

The goal of DR as a computational problem is to detect whether a given drug and disease pair has a therapeutic association. There are different approaches that have the potential to accelerate the drug discovery process and reduce costs. These methods can be classified into three main categories called feature-based (FB), heterogeneous network-based (HNB), and knowledge graph-based (KG) models. Table 1 provides an overview of current studies on the DR problem. In FB approaches, various features of drugs and diseases are extracted to predict drug-disease associations [3–8]. Given that FB methods fail to illustrate the correlation between features, it was necessary to consider HNB models as an alternative. HNB models are improved to use the relationship between features for finding drug-disease associations [9–16]. However, these methods are unable to identify the types of relationships between features. Recently, researchers have been interested in the KG approaches for predicting drug-disease associations. To elaborate, KGs are a proper way to represent information and knowledge in a structural foundation. In order to create a KG, it is essential to address these fundamental aspects: KG construction, node and relationship definition (edge types), and numerical vector embedding, which all play a vital role in its effectiveness. A knowledge graph as $G=<V,E,R>$ is defined where $V$ shows the set of nodes, $E$ demonstrates the set of edges, and $R$ is the relationship types between nodes. In KG, each triplet is specified as $<h, r, t> \in E$, where $h,t\in V$, and $r\in R$ shows the relationship type $r$ between $h,t.$.

**Table 1:** An overview of current studies in the drug repurposing field

Reference	Method	Details
[3]	FB	Non-linear method
[4]	FB	Fusion method to combine three similarity measurements
[5]	FB	Recommendation system based on functional characteristics
[9]	HNB	Non-negative matrix factorization
[10]	HNB	Multi-modal neural network
[11]	HNB	Random walk with restart on a hierarchical network
[12]	HNB	Weighted bilinear neural network
[13]	HNB	Deep learning
[14]	HNB	Geometric deep learning
[15]	HNB	Fusing higher and lower-order biological information
[17]	KG	Creating a knowledge graph from biomedical literature
[19]	KG	Literature-based KG utilizing weighted relationships
[20]	KG	Drug-centric knowledge graph
[21]	KG	A coupled-tensor factorization-based embedding model
[23]	KG	CAS biomedical knowledge graph and drug ranking method
[18]	KG	Knowledge graph completion using TransE
[22]	KG	Logistic regression method

Numerous studies have focused on developing efficient knowledge graphs by incorporating nodes and relationships that are relevant, informative, and sufficient. Some methods [17–19] extract abstracts of a huge number of articles and, after tokenizing them, make a graph using relationships between the tokens. While these methods perform well, they require complex computations and powerful computer systems to prepare the embeddings and then predict possible drug-disease associations due to the large volume of data [17–19]. Alternatively, some methods create the customized KGs by selecting specific node types, such as drugs, targets, and diseases, and relationship types, such as drug-target and drug-disease associations, based on their research objectives [20–23]. However, selecting the right feature set remains a challenge with these methods. Some of them emphasize the drug features (known as drug-centric KG) [20], while others consider three main entity types: drugs, diseases, and genes, with most relationships defined on the gene features [21, 23]. While gene-based relationships are more abundant, drug-disease associations are scarcer, which may mislead the embedding techniques used by these methods for drug-disease association predictions. Therefore, it is important to carefully consider the entity types and relationships included in KGs to ensure accurate predictions.

One of the most recent customized KG-based methods is DrugRep-KG [22]. The method constructs a drug-disease KG (DDKG) based on the drugs, diseases, and their features using 11 node types and 10 relationship types. It even includes the chemical structure of drugs, while the aforementioned methods have not considered it. Unlike drug-centric KGs [20], DrugRep-KG incorporates disease features in addition to drug information. Moreover, the list of intended features for diseases is more informative than previous studies [21, 23] by making use of MeSH terms and semantic types. Furthermore, the advantage of DrugRep-KG over the mentioned methods is that it simplifies relationships and reduces the complexity of paths among drugs and diseases, making it better able to capture potential drug-disease associations [22]. Later, to represent the entities and relationships of DDKG, DrugRep-KG employs Word2Vec as a natural language processing technique, creating numerical vectors for each entity and relationship. While subtracting the vectors of related drugs and diseases does not always result in close proximity, DrugRep-KG utilizes concatenation of drug and disease vectors as an alternative approach for feeding the logistic regression model. This approach allows the DrugRep-KG method to predict new therapeutic indications for existing drugs.

It is possible that the reason for the inconsistency observed when subtracting vectors instead of concatenating them lies in the construction and embedding of the DDKG. Additionally, the DrugRep-KG method does not consider possible intra-relationships among drug features and disease features, nor does it take into account possible inter-relationships between drug and disease features. This suggests that the method may not be as efficient at identifying potential drug candidates for a specific disease as it could be and highlights the need for improvements in these areas.

This paper presents the DrugRep-HeSiaGraph model, inspired by DrugRep-KG [22]. By adding more types of relationships, such as protein–protein interactions, gene interaction networks, protein-domain relationships, and protein-gene relationships, the proposed model makes it easier to find drug candidates for diseases. In addition, it employs Word2Vec to represent the DDKG. Despite the existence of various methods for embedding DDKG, such as TransE [24], TransR [25], TransH [26], Node2Vec [27], ComplEx[28], and others, a challenge arises due to the presence of 11 relationship types in a 'Many-to-Many' configuration. It is important to note that Node2Vec and TransE are suitable for 'One-to-One' and ‘One-to-Many’ relationship types and cannot consider multiple relationship types in ‘Many-to-Many’ form. On the other hand, TransR and TransH generate distinct embedding vectors for each node based on every relationship type and fail to make an embedding considering all relationship types. Similarly, even though ComplEx handles multiple relationship types in ‘Many-to-Many’ configuration, it requires complex networks, making it computationally expensive. Therefore, in this study, we utilize Word2Vec to generate embeddings that considers multiple relationship types in a 'Many-to-Many' configuration. This approach is computationally faster and is capable of capturing complex relationships between entities and relationships. To do so, we implement the continuous bag-of-words (CBOW) architecture of Word2Vec [29]. CBOW embeds each node based on its context, taking into account both local relationships and global relationships that reflect the similarities and distances of nodes beyond direct connections. These global relationships are often derived from higher degree relations. Furthermore, DrugRep-HeSiaGraph proposes a heterogeneous siamese neural network (SNN), called HeSiaNet, for bringing related drugs and diseases presentations into a more accurate unified latent space. HeSiaNet is a type of dual-channel network that consists of two network channels and a similarity learning component with various weights. The siamese networks, as described by Chicco [30], allow the network to learn similarities between input pairs by comparing the features extracted from each channel.

The DrugRep-HeSiaGraph method is evaluated by comparing its performance with DrugRep-KG in different settings, DisDrugPred as a matrix factorization method, and DRP-VEM as an ensemble method. Furthermore, we discuss the effectiveness of our model in suggesting drugs for coronavirus infection as a disease that has no known associations with drugs. This evaluation aims to assess the model's ability to tackle new diseases. The main contributions of DrugRep-HeSiaGraph are briefly listed as follows:

- Incorporating a DDKG named DDKG-V1 by protein–protein interactions, gene interaction networks, protein-domain relationships, and protein-gene relationships,
- Designing a heterogenoeus SNN, named HeSiaNet, for predicting the drug-disease associations.

## Methods

In this section, we aim to build a computational model that helps us forecast the potential treatments for a given disease to reduce drug development time, cost, and risk. To do so, we introduce a method to learn an embedding space with lower dimensionality for drugs and diseases. This section explains the DR problem and the proposed model to address it.

### Drug repurposing problem

**Fig.1:**
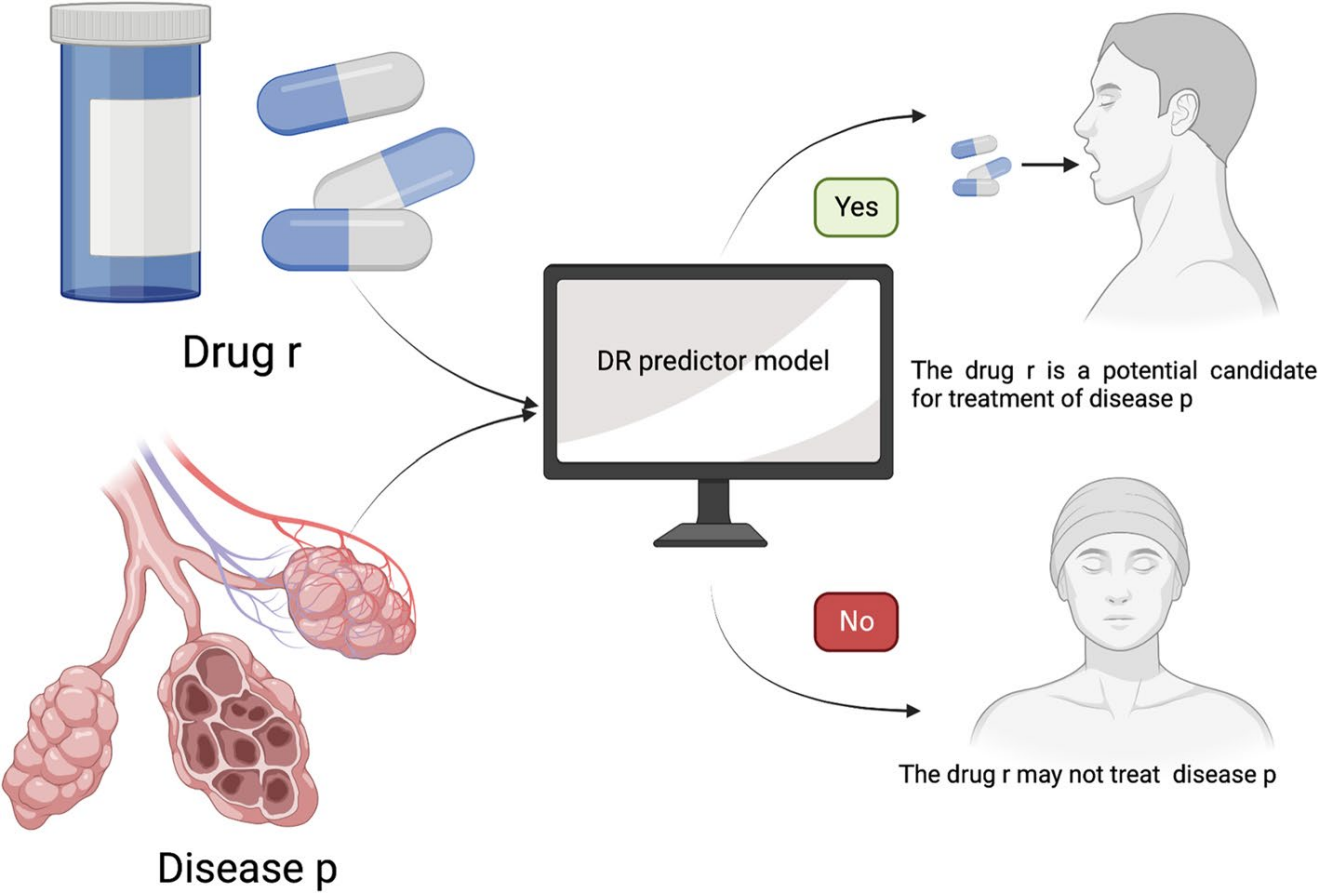
The inputs and outputs of the drug repurposing (DR) problem

The sets of diseases and drugs are shown by ${\varvec{\rho}}=\{{{\varvec{p}}}_{1},{{\varvec{p}}}_{2},\dots ,{{\varvec{p}}}_{{\varvec{n}}}\}$ and $\boldsymbol{\varphi }=\{{{\varvec{r}}}_{1},{{\varvec{r}}}_{2},\dots ,{{\varvec{r}}}_{{\varvec{m}}}\}$, respectively, where $|{\varvec{\rho}}|={\varvec{n}}$ and $\left|\boldsymbol{\varphi }\right|={\varvec{m}}$. DR problem aims to predict whether drug ${\varvec{r}}\in \boldsymbol{\varphi }$ is prescribed for disease $p \in \rho$ or not. So, the model’s input is a drug and disease pair, and the output is one if the drug treats the disease and zero otherwise. Figure 1 illustrates the problem schematically.

### DrugRep-HeSiaGraph method

**Fig. 2:**
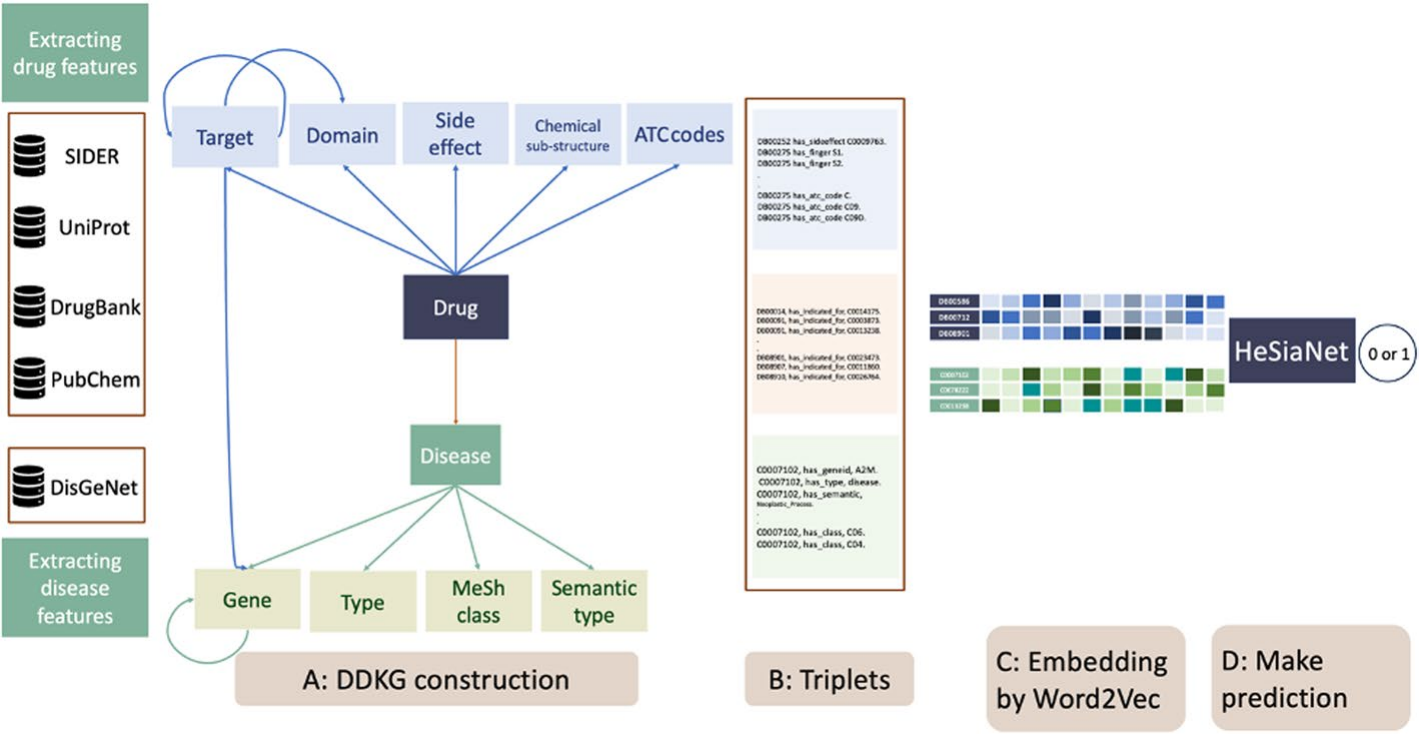
DrugRep-HeSiaGraph method

**Fig. 3:**
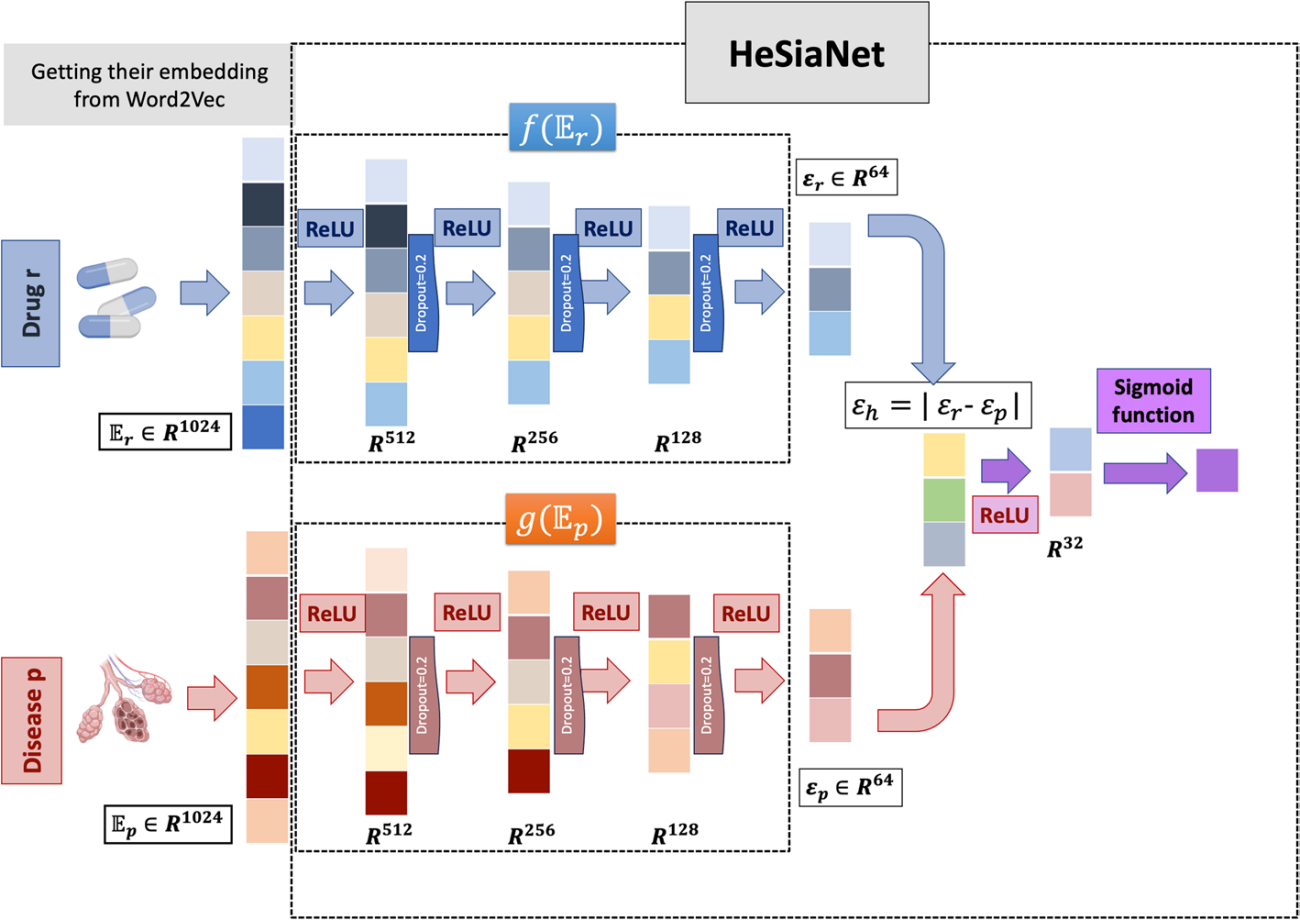
HeSiaNet architecture

The DrugRep-HeSiaGraph method consists of two main steps to address DR problem as follows:

- Drug-disease knowledge graph step
  - Constructing a drug-disease knowledge graph named DDKG-V1 (see Fig. 2-A),
  - Embedding the entities of DDKG-V1 (see Figs. 2-B and 2-C),
- Heterogeneous siamese neural network step
  - Designing a heterogeneous SNN, called HeSiaNet, to predict drug-disease association (see Fig. 2-D and Fig. 3)

#### Drug-disease knowledge graph step

This section describes the construction process of the DDKG in DrugRep-HeSiaGraph, called $\text{DDKG-V1}=<V, E,R>$. There are several features that can be applied to the model, including, but not limited to, symptoms, signs, gene expression profiles, molecular pathways, genomics, and more. While incorporating all these data types can be valuable, there are certain obstacles to consider. Firstly, it is necessary to identify features that are consistently available for all drugs and diseases in order to ensure comprehensive coverage. Secondly, the chosen features should be compatible with the graph-based representation employed in our model. To do so, we select chemical structure, target, domain, side effect, and anatomical therapeutic chemical (ATC) code as drug features, and genes, MeSH class, type, and semantic type as disease features. Therefore, DDKG-V1 includes 11 node types (see Table 2). The six node types pertain to drugs, displaying the drug names and five drug features. Meanwhile, the other five node types relate to diseases, indicating the disease names and four disease features. The node set of DDKG-V1 is comprised as follows:

1 $$V = \varphi \cup C \cup T \cup D \cup S \cup A \cup \rho \cup {\mathbb{T}} \cup {\mathbb{C}} \cup {\mathbb{G}} \cup {{\mathbb{S}} }.$$

**Table 2:** Stochastic of the applied dataset

Relationship	Entity	Node types	Relationship type	Database	The node numbers	The number of relationships
Intra-relationships	Drug	Name ($\varphi$)	–	DrugBank [35]	410	–
		Chemical Structure ($C$)	has_chemical_substructure	PubChem [31]	881	52,979
		Target Protein ($T$)	has_target has_interaction	DrugBank [35] STRING [36]	1506	2122 6909
		Protein Domain ($D$)	has_domain has_domain_target	UniProt [37]	1070	1828 2804
		Side Effect ($S$)	has_side_effect	SIDER4.1 [38]	5734	64,121
		ATC Code ($A$)	has_ATC_code	SIDER4.1 [38]	1087	2958
	Disease	Name ($\rho$)	–	DisGeNET [39]	141	–
		Type (${\mathbb{T}}$)	has_type	DisGeNET [39]	3	141
		Class (${\mathbb{C}}$)	has_class	DisGeNET [39]	22	9440
		Gene (${\mathbb{G}}$)	has_gene has-gene-interaction	DisGeNET [39] STRING [36]	3561	5891 16,870
		Semantic (${\mathbb{S}}$)	has-semantic-type	DisGeNET [39]	5	141
Inter-relationships		Target-Gene	has_encoded	UniProt [37]		633
		Drug-Disease	has_treatment	repoDB [40]		748

Moreover, it contains 14 relationship types, which are divided into three groups as follows:

- Intra-relationship types for drugs, which indicate among drug features,
- Intra-relationship types for diseases, which show among disease features,
- Inter-relationship types for drugs and diseases that depict relationships between drug and disease features.

The details of these relationship types ($R$) to construct DDKG-V1 are introduced as below (see Table 2):

1. Intra-relationship types for drugs
  1.1 if drug $r\in \varphi$ has a chemical substructure such as $c\in C$ extracted from a PubChem fingerprint [31], its relationship is shown by “*r* has_chemical_substructure $c$”,
  1.2 if drug $r\in \varphi$ is targeted by a protein such as $t\in T$, then its relationship is represented by “*r* has_target $t$” [32, 33],
  1.3 if drug $r\in \varphi$ is targeted by protein *t*, where this protein has a domain such as $d\in D$, then its relation is displayed by “*r* has_domain $d$”,
  1.4 if drug $r\in \varphi$ has a side effect such as $s\in S$, then its relationship is illustrated by “*r* has_side-effect *s*” [34],
  1.5 if drug $r\in \varphi$ has an ATC code such as $a\in A$, then its relationship based on each code level is shown by “*r* has_ATC_code *a*”,
  1.6 if proteins $t,t{\prime}\in T$ have physical interaction, then its relationship is represented by “*t* has_interaction $t{\prime}$,
  1.7 if protein $t\in T$ has a domain such as $d\in D$, then its relationship is displayed by “*t* has_domain_taregt_relation *d*”.
2. Intra-relationship types for diseases
  2.1 if the type of disease $p\in \rho$ is ${\mathbb{t}}\in {\mathbb{T}}$, so its relationship is illustrated by “*p* has_type ${\mathbb{t}}$”,
  2.2 if the class of disease $p\in \rho$ is ${\mathbb{c}}\in {\mathbb{C}}$, then its relationship is represented by “*p* has_class ${\mathbb{c}}$”,
  2.3 if gene ${\mathbb{g}}\in {\mathbb{G}}$ is associated with disease $p\in \rho$, then its relationship is conducted as “*p* has_gene ${\mathbb{g}}$”,
  2.4 if the standard categorization of disease $p\in \rho$ is ${\mathbb{s}}\in {\mathbb{S}}$ based on the unified medical language system (UMLS), then its relationship is demonstrated by “*p* has-semantic-type ${\mathbb{s}}$”,
  2.5 if genes ${g},{g'}\in {\mathbb{G}}$ have interaction to show their functional relationship, then their relationship is shown by “${g}$ has_gene_interaction *g’*”.
3. Inter-relationship types for drugs and diseases
  3.1 If drug $r\in \varphi$ treats disease $p\in \rho$, then their relationship is displayed by “*r* has-treatment *p*”.
  3.2 if gene ${\mathbb{g}}\in {\mathbb{G}}$ encodes the target$t\in T$, then their relationship is represented by “${\mathbb{g}}$ has_encoded *t*”,

Finally, the edge set of DDKG-V1 is prepared as below:

2 $$E = \left\{ {\left\langle {v, r,v^{\prime } } \right\rangle |v,v^{\prime } \in V \& r \in R, \;{\text{node}}\;v\;{\text{and}}\;v^{\prime } \;{\text{are}}\;{\text{related}}\;{\text{based}}\;{\text{on}}\;{\text{relationship}}\;{\text{type}}\;r} \right\}.$$

We present the DDKG-V1 as evidence of triplet sentences, similar to Eq. 2, and then feed it into the CBOW-based Word2Vec implementation to construct nodes and relationships embeddings. The corresponding vector for a node $v\in V$ is represented by ${\mathbb{E}}_{v}$.

#### Heterogeneous siamese neural network step

Recall that we propose to learn the representation of data in a unified latent space. Inspired by retrospective studies [7, 10], we leverage the power of deep neural networks to address the DR problem and suggest using the advantage of heterogenous SNN. This section describes the details of the proposed heterogeneous SNN, HeSiaNet, which aims to bring associated drugs and diseases closer together in a lower dimensional space and predicts whether the given drug-disease pairs are associated (1) or not (0). Figure 3 shows the proposed model architecture.

In the following, the main steps of the HeSiaNet are provided, and later these steps are explained.

1. Getting the embedding vector of drug $r$ and disease $p$ using Word2Vec based on DDKG-V1 presented by ${\mathbb{E}}_{r}$ and ${\mathbb{E}}_{p}$, respectively.
2. Feeding ${\mathbb{E}}_{r}$ to function $f({\mathbb{E}}_{r})$, which consists of an input layer, three hidden layers activated by ReLU and using dropout, and an output layer, which presents the embedding of drug $r$ in unified latent space shown by ${\varepsilon }_{r}$.
3. Feeding ${\mathbb{E}}_{p}$ to function $g({\mathbb{E}}_{p})$, which consists of an input layer, three hidden layers activated by ReLU and using dropout, and an output layer, which presents the embedding of disease $p$ in unified latent space presented by ${\varepsilon }_{p}$.
4. Absolute subtracting the outputs of functions $f\left( {{\mathbb{E}}_{r} } \right)$ and $g\left( {{\mathbb{E}}_{p} } \right)$ to brings associated drugs and diseases closer in the latent space and produce $\varepsilon_{h} = \left| {\varepsilon_{r} - \varepsilon_{p} } \right|$.
5. Feeding $\varepsilon_{h}$ to a dense hidden layer with ReLU activation function and then using a sigmoid function to predict the probability of association.
6. If the probability is greater than 0.5, the drug and disease are considered to be associated; otherwise they are not.

HeSiaNet comprises two distinct channels: the drug embedding channel (shown by $f\left( {{ }{\mathbb{E}}_{r} } \right)$ based on ${\mathbb{E}}_{r}$ as the embedding of drug $r \in \varphi$), and the disease embedding channel (shown by $g\left( {{\mathbb{E}}_{p} } \right)$ based on ${\mathbb{E}}_{p}$ as the embedding of disease $p \in \rho$), to predict the therapeutical association between drug $r$ and disease $p$. The reason for employing a heterogeneous instead of a homogeneous SNN is that drugs and diseases have different natures and are not of the same type. Thus, we necessitate two distinct networks that are optimized concurrently, albeit with differing weight updates. In other words, the extracted embeddings of drugs and diseases from the DDKG-V1 are numerical vectors (${\mathbb{E}}_{r}$ and ${\mathbb{E}}_{p}$), and $f\left( {{ }{\mathbb{E}}_{r} } \right)$ and $g\left( {{\mathbb{E}}_{p} } \right)$ project these vectors onto a unified latent space, allowing drugs and diseases to be compared and analyzed based on their shared properties.

More specifically, each channel learns a non-linear function on the representation of the drugs and diseases, extracted from DDKG-V1, called $f\left( {{\mathbb{E}}_{r} } \right):R^{k} \to { }R^{h}$ and $g\left( {{\mathbb{E}}_{p} } \right):R^{k} \to { }R^{h}$, respectively. The architecture of $f\left( {{\mathbb{E}}_{r} } \right)$ is based on several dense layers that produce ${\upvarepsilon }_{r}$ as the lower-dimensional representation ($h$-dimension) of drug $r$. Similarly, $g\left( {{\mathbb{E}}_{p} } \right)$ is defined to prepare $\varepsilon_{p}$ as the representation of diseases $p$ in the $h$-dimensional space. Since the SNNs are commonly used for tasks that involve similarity or distance measurement between two inputs, the aim here is to bring drugs and diseases with therapeutical associations closer together and vice versa. For this purpose, we perform an element-wise subtraction of the vectors ${\upvarepsilon }_{r}$ and $\varepsilon_{p}$ to generate a new vector $\varepsilon_{h} = \left| {\varepsilon_{r} - \varepsilon_{p} } \right|$. The resulting vector undergoes processing through several dense hidden layers of lower sizes before making the final prediction via a sigmoid function. Since the sigmoid function outputs a probability of association between drug $r$ and disease $p$, which lies in the range [0,1], we consider a probability greater than 0.5 to indicate association, and otherwise, it is considered to be not association. However, to minimize the difference between the predicted probability distribution and the actual probability distribution, we define the binary-cross entropy loss function [41] for the final layer of the network, calculated as below:

3 $$L\left( {y_{r,p} ,{ }\widehat{{y_{r,p} }}} \right) = - \left[ {y{*}\log \left( {\widehat{{y_{r,p} }}} \right) + \left( {1 - y_{r,p} } \right){*}\log \left( {1 - \widehat{{y_{r,p} }}} \right)} \right],$$

where, $y_{r,p}$ indicates the actual state of association between drug $r$ and disease $p$**,** and $\widehat{{y_{r,p} }}$ shows the predicted association state. The details of the architecture of HeSiaNet are illustrated in Fig. 3 and explained in section “HeSiaNet architecture and training procedure”.

## Results and discussion

To assess the performance of our proposed method, DrugRep-HeSiaGraph, several experiments based on multiple criteria are conducted. This section encompasses the preparation of the dataset and the setting of hyperparameters, as well as a detailed explanation of the HeSiaNet architecture and its training procedure. The evaluation criteria employed for the model are also described, followed by a comparison of the results obtained using DrugRep-HeSiaGraph and the state-of-the-art models, DrugRep-KG, DisDrugPred and DRP-VEM in two ways: assessment of their input representation and evaluation of their performances. Furthermore, this section investigates the efficiency of applying homogeneous SNN (Hom-SNN) compared to heterogeneous SNN (HeSiaNet). Finally, the practical application of the DrugRep-HeSiaGraph model in addressing real-world challenges in the field of drug repurposing is highlighted by suggesting treatments for COVID-19.

### Dataset preparation

The basic dataset is generated as below:

4 $$X = \{ \left\langle {r,{ }p} \right\rangle |r \in \varphi { }\& { }p \in \rho ,{\text{ and}}\left\langle {r,p} \right\rangle \in {\mathfrak{A}} \cup {\mathfrak{E}}\} ,$$

where ${\mathfrak{A}}$ and ${\mathfrak{E}}$ are considered, respectively, as positive data (known drug-disease association) and negative data (unknown drug-disease association with respect to diseases that are in common with adverse drug reactions), as follows:

5 $${\mathfrak{A}} = \left\{ {\left\langle {r,p} \right\rangle {| }r \in \varphi { }\& { }p \in \rho ,\;{\text{drug}}\;r\;{\text{has}}\;{\text{been}}\;{\text{indicated}}\;{\text{for}}\;{\text{disease}}\;p{ }} \right\},$$

6 $${\mathfrak{E}} = \left\{ {\left\langle {r,p} \right\rangle |r \in \varphi \;\& \;p \in \rho ,\;{\text{drug}}\;r\;{\text{causes}}\;{\text{disease}}\;p\;{\text{because}}\;{\text{of}}\;{\text{adverse}}\;{\text{reaction}}} \right\}.$$

Furthermore, the label of each $x_{r,p} \in X$ is denoted as below:

7 $$y_{r,p} = \left\{ {\begin{array}{*{20}c} {1 \left\langle {r,p} \right\rangle \in {\mathfrak{A}}, } \\ { 0 \left\langle {r,p} \right\rangle \in {\mathfrak{E}} .} \\ \end{array} } \right.$$

Moreover, the known drug-disease associations (${\mathfrak{A}})$ are gathered by utilizing the repoDB[40] database, which is a database dedicated to drug repositioning. Initially, the associations are filtered based on their 'approved' status to ensure training the model with FDA-approved drug-disease associations. Subsequently, the lists of drugs and diseases are narrowed down based on the availability of all the relevant features. To create the negative set, we consider unknown drug-disease associations in which the disease corresponds to an adverse drug reaction. The hypothesis is based on the idea that if a drug causes any side effect, that is in common with our disease set, it cannot treat that, limiting its usefulness for that particular disease [22]. The statistics of the applied dataset are available in Table 3.

**Table 3:** Statistics of dataset

	# Drugs	# Diseases	# Drug-Disease pairs
Positive dataset ($\left| {\mathfrak{A}} \right|$)	410	141	748
Negative dataset ($\left| {\mathfrak{E}} \right|$)	361	89	1966
Basic dataset ($\left| X \right|$)	410	141	2714

We extract 10% of the positive data (74 pairs of ${\mathfrak{A}}$) and an equal number of negative data (74 pairs of ${\mathfrak{E}}$) to create the test set. To prevent Word2Vec from considering the therapeutic or non-therapeutic associations between drug-disease pairs in the test set, these relationships are removed while creating the versions of DDKG.

To demonstrate that Word2Vec effectively captures the heterogeneity of DDKG-V1 while generating the embeddings from multiple node types and relationship types, we conduct an experiment to visualize them in a lower-dimensional space. Accordingly, the t-SNE ( t-distributed Stochastic Neighbor Embedding) technique is employed [42] as a popular dimension reduction technique that visualizes data with a high dimension in a lower one. This technique is also helpful for revealing the patterns in the data [42].

**Fig. 4:**
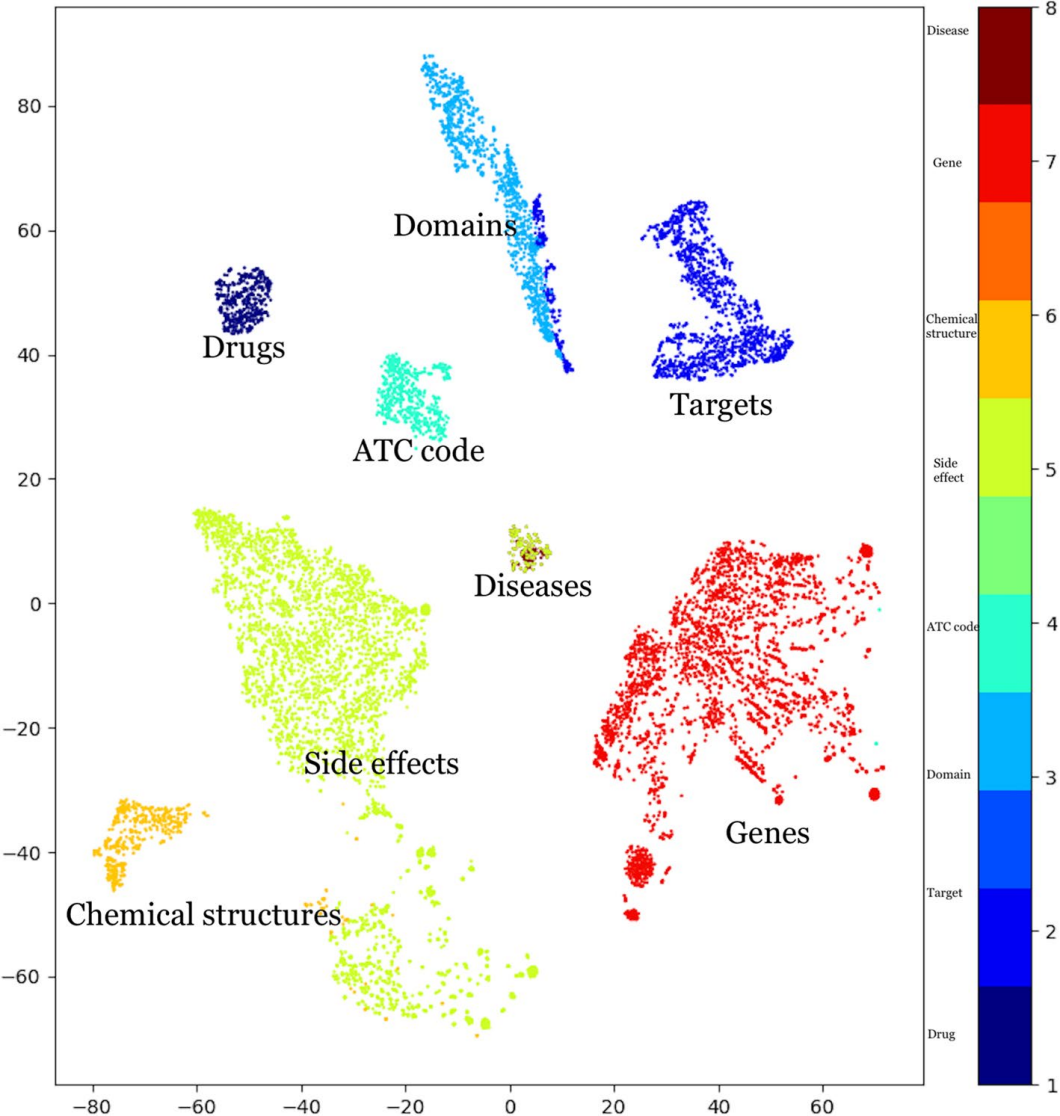
The t-SNE distribution for entities of DDKG-V1 embedded by Word2Vec

The t-SNE plot is illustrated in Fig. 4 and generated based on embeddings of DDKG-V1’s entities, which are labeled according to their types (drug, disease, target, domain, side effect, etc.). The t-SNE plot indicates that different node types are clustered separately, demonstrating the effectiveness of Word2Vec in capturing the semantic relationships among entities. Figure 4 illustrates that target and domain entities, which share a close conceptual relationship as mentioned earlier, are also positioned in close proximity in the t-SNE plot. Notably, the ATC codes, which encode anatomical, therapeutic, and chemical information about drugs and are closely related to drug treatment mechanisms, form a cluster that bridges the gap between the drug and disease clusters. Furthermore, the plot reveals the presence of common members between the side effects and diseases, as evident from their overlapping cluster.

By visually analyzing the t-SNE plot, we can observe the distinct clusters formed by different node types and gain insights into their relative positions. This visualization reinforces the presence of heterogeneity in our data and provides a valuable perspective on the relationships between various entities within our DDKG-V1.

Moreover, to address the issue of imbalanced data, we investigate different strategies, mainly oversampling, under-sampling and a combination of over-sampling and under-sampling techniques. The experiment results show the combination approach obtains the best performance (see Table 4). Finally, the model is evaluated based on five-fold cross-validation.

### Evaluation criteria

To evaluate the performance of DrugRep-HeSiaGraph, we employ five evaluation criteria: accuracy (ACC), area under the receiver operating characteristic curve (AUC-ROC), area under the precision-recall curve (AUC-PR), brier score (BS), Matthew’s correlation coefficient (MCC), and F1-score.

ACC measures the proportion of correct predictions out of all predictions and is calculated as below:

8 $$ACC = \frac{TP + TN}{{TP + TN + FP + FN}},$$

where, the TP, TN, FP, and FN indicate true positive, true negative, false positive and false negative, respectively.

The AUC-ROC [43] score quantifies the area under the receiver operating characteristic curve, which plots the true positive rate (TPR) against the false positive rate (FPR) at different cut-offs. TPR and FPR are defined as below:

9 $$TPR = \frac{TP}{{TP + FN}},{ }FPR = \frac{FP}{{FP + TN}}.$$

The AUC-PR [44] computes the area under the precision-recall curve, which plots precision against recall at different thresholds. Precision and recall scores are defined as follows:

10 $$Precision = \frac{TP}{{TP + FP}},{ }Recall = \frac{TP}{{TP + FN}}.$$

The BS [45] is a scoring rule to evaluate the accuracy of probabilistic predictions. It calculates the mean square difference between the predicted and actual outputs. Therefore, the smaller BS, the better the performance of the model. This score is measured as follows:

11 $$BS = \frac{1}{N}\mathop \sum \limits_{l = 1}^{N} \left( {y_{{\left( {r,p} \right)l}} - \widehat{{y_{{\left( {r,p} \right)l}} }}} \right)^{2}$$

where the $N$ is the number of instances of the test set, $y_{{\left( {r,p} \right)l}}$ indicates the actual status of association for the $l_{th}$ sample of the test set, and $\widehat{{y_{{\left( {r,p} \right)l}} }}$ shows the predicted likelihood of association.

The MCC is a measurement for assessing the quality of binary classification methods and is widely used in bioinformatics problems [46] and is calculated accordingly:

12 $$MCC = \frac{TP.TN - FN.FP}{{\sqrt {\left( {TP + FP} \right).\left( {TP + FN} \right).\left( {TN + FP} \right).\left( {TN + FN} \right)} }}.$$

Finally, the F1-score, as the last considered criterion, merges precision and recall (see Eq. 10) using harmonic mean and generates a unified metric for assessing the models. This criterion is mostly used when the dataset is imbalanced. F1- score is expressed as follows:

14 $$F1 - score = \frac{Precision*Recall}{{Precision + Recall}}.$$

### Hyper parameter setting

The proposed model is developed using Python 3.9 and utilizes the Gensim [47], Keras [48], and imbalanced-learn [49] packages. The Gensim package [47] is used to generate the embedded vector of drugs and diseases based on the CBOW implementation, with the *vector_size* parameter set to 1024 using a grid search method. The Keras package [48] is employed to create the HeSiaNet model. To address the dataset imbalance, we apply the imbalanced-learn package [49] using five different strategies: using an imbalanced dataset for training (IMB), randomly under sampling negative pairs (RUS), randomly oversampling positive pairs (ROS), oversampling positive pairs using the SMOTE technique (OSMOTE), and a combination of oversampling by SMOTE and random under sampling (SORU), as suggested by previous studies [50]. According to Table 4, the best results are achieved by oversampling positive pairs by 90% using the SMOTE technique followed by randomly under sampling negative pairs (SORU).

**Table 4:** The comparison of applied strategies for facing imbalanced dataset challenge

Strategy	ACC	AUC-ROC	AUC-PR	BS	MCC	F1-score
IMB	80.64	**92.69**	**91.69**	12.94	62.76	84.21
RUS	83.96	92.48	91.56	8.25	68.07	84.14
ROS	85.72	92.61	90.76	10.92	71.61	82.67
OSMOTE	84.62	92.19	90.49	11.18	69.34	82.07
SORU	**86.38**	92.57	91.55	**10.69**	**73.02**	**84.23**

### HeSiaNet model architecture and training procedure

This paper presents the DrugRep-HeSiaGraph, which uses a heterogeneous SNN, named HeSiaNet, with two different channels to explore the relationship between drug-disease pairs. The choice of a heterogeneous network is appropriate because drugs and diseases have distinct natures and come from different categories.

The input of the HeSiaNet model consists of drug and disease vectors with a length of 1024. The drug channel layers are defined based on the drug vector, while the disease channel layers are optimized using the disease vector. Each channel of the network comprises four dense layers with 512, 256, 128, and 64 neurons, respectively, with ReLU serving as the activation function for all the dense layers. Dropout regularization with a rate of 0.2 is applied to each dense layer to prevent overfitting. Given the goal of bringing similar concepts closer in the latent space, it is important to ensure that the distributions of the embeddings are close. In this manner, the KL-divergence loss function is imposed for both channels. KL-divergence, or Kullback–Leibler divergence, is measured to calculate the difference between two probability distributions [51], and it is calculated as below:

15 $$D_{KL} \left( {f,{ }g} \right) = \smallint {\text{log}}\left( {\frac{{f\left( {{\mathbb{E}}_{r} } \right)}}{{g\left( {{\mathbb{E}}_{p} } \right)}}} \right)$$

Then, the output of the two channels is subtracted ($\varepsilon_{h}$) and is subsequently passed through a final dense layer with 32 neurons and a ReLU activation function, with dropout regularization of 0.2 applied to this layer. The resulting vector is then connected to a single dense layer with a sigmoid activation function to yield the final prediction of the network.

The HeSiaNet is trained utilizing the Adam optimizer with a learning rate of $5{ }e - 5$. During training, the model is monitored with the validation set, and the training is stopped early if the validation accuracy does not improve for a certain number of epochs using the 'EarlyStopping' callback. Additionally, the learning rate is reduced using the 'ReduceLROnPlateau' callback if the validation accuracy does not improve for a certain number of epochs. Finally, the best weights obtained during training are saved using the 'ModelCheckpoint' callback. The use of different channels for drugs and diseases, along with the Adam optimizer and various callbacks for training, contributes to the HeSiaNet’s effectiveness and accuracy.

### Evaluating the performance of DrugRep-HeSiaGraph

To assess the performance of our model, we use a technique called five-fold cross-validation, a technique that divides the dataset into five subsets (or “folds”). The model is trained on four of these folds and evaluated on the remaining one. We repeat this process five times, ensuring that each subset serves as the test set once.

Additionally, to account for potential variations in model performance due to the selected validation set, each experiment is conducted four times. As a result, DrugRep-HeSiaGraph consistently demonstrates strong performance in this evaluation, boasting an average AUC-ROC of 91.16%, an AUC-PR of 90.32%, an ACC rate of 84.63%, a BS score of 0.119, and an MCC score of 69.31%. These outcomes provide strong evidence of our model's effectiveness in predicting drug-disease associations.

### Analysis of features

The objective of this section is to examine drug features, such as chemical structure, target, domain, side effect and ATC code, alongside diseases features, including genes, semantic type, MeSH class, and type. We aim to identify the features that exert the greatest influence on the performance of the proposed model and are crucial to consider in other studies related to DR problem.

To achieve this, we conduct two types of experiments: drug-based and disease-based. Drug-based experiments incorporate all the disease features while experimenting with various combinations of drug features. Similarly, in disease-based experiments, we employ all drug features while exploring different combinations of disease-related features.

We consider the most crucial experiments to be those where the model is exclusively trained with a single feature, and where the model incorporates all features except one. The purpose behind removing each feature is to discern which one exerts the most significant impact on the model’s performance, leading to the greatest reducing in the base model’s effectiveness. Conversely, when we retain only one feature, our objective is to determine which feature contributes the most valuable information, resulting in a minimal deviation from the base model that employs all features. Furthermore, we explore various possible combinations of drug or disease features with the corresponding model scores detailed in Table S1 of the appendix. Our analysis demonstrates that using all features is advisable, as it yields the best scores using DDKG-V1. These findings underscore the importance of incorporating all features to enhance the accuracy of predictions.

**Fig. 5:**
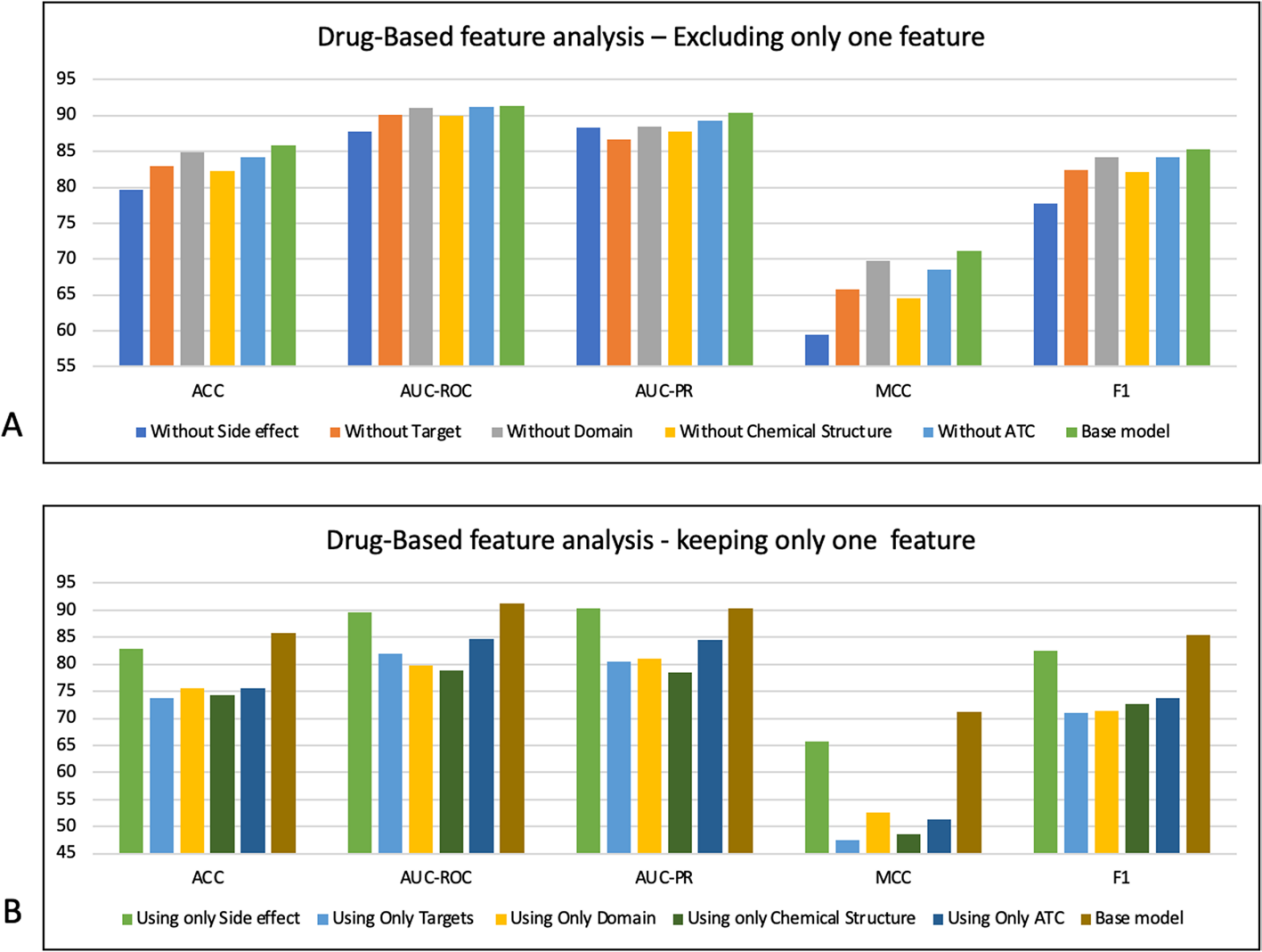
Drug-based feature analysis

Regarding the drug-based feature analysis, Fig. 5 provides an overview of the model's performance when one drug feature is omitted (A) or when only one drug feature is included (B). As depicted in Fig. 5-A, the exclusion of the side effect feature (dark blue bar) results in a significant decline in the base model's performance (green bar). For instance, the removal of side effects as a feature leads to a decrease of approximately 6% in the ACC and 8% in the F1-score. Conversely, when only the side effect is employed as the sole drug feature (see Fig. 5-B, the light green bar), the model's performance exhibits a less pronounced changes compared to the base model (brown bar). In this case, the ACC decreases by 3%, and the F1-score reduces by approximately 6%. In contrast, utilizing only one of the other drug features results in a more substantial reduction, with approximately a 10% decrease in ACC and a 20% drop in the F1-score. Additionally, the combination of drug features that included the side effect yield better scores (as shown in Table S1).

These findings highlight the significance of side effects as a crucial drug feature in DR problems. Intriguingly, our previous research [16] also identified side effect as the most important feature for drug repurposing. Other studies have also investigated the efficacy of side effects as a significant feature in addressing DR problem [52, 53]. Additionally, biological studies have demonstrated that the shared side effects among drugs can indicate a common molecular function. This mechanism elucidates how drugs with similar side effects can effectively treat the comparable diseases [52]. These findings confirm the necessity of including side effects feature in DR studies.

**Fig. 6:**
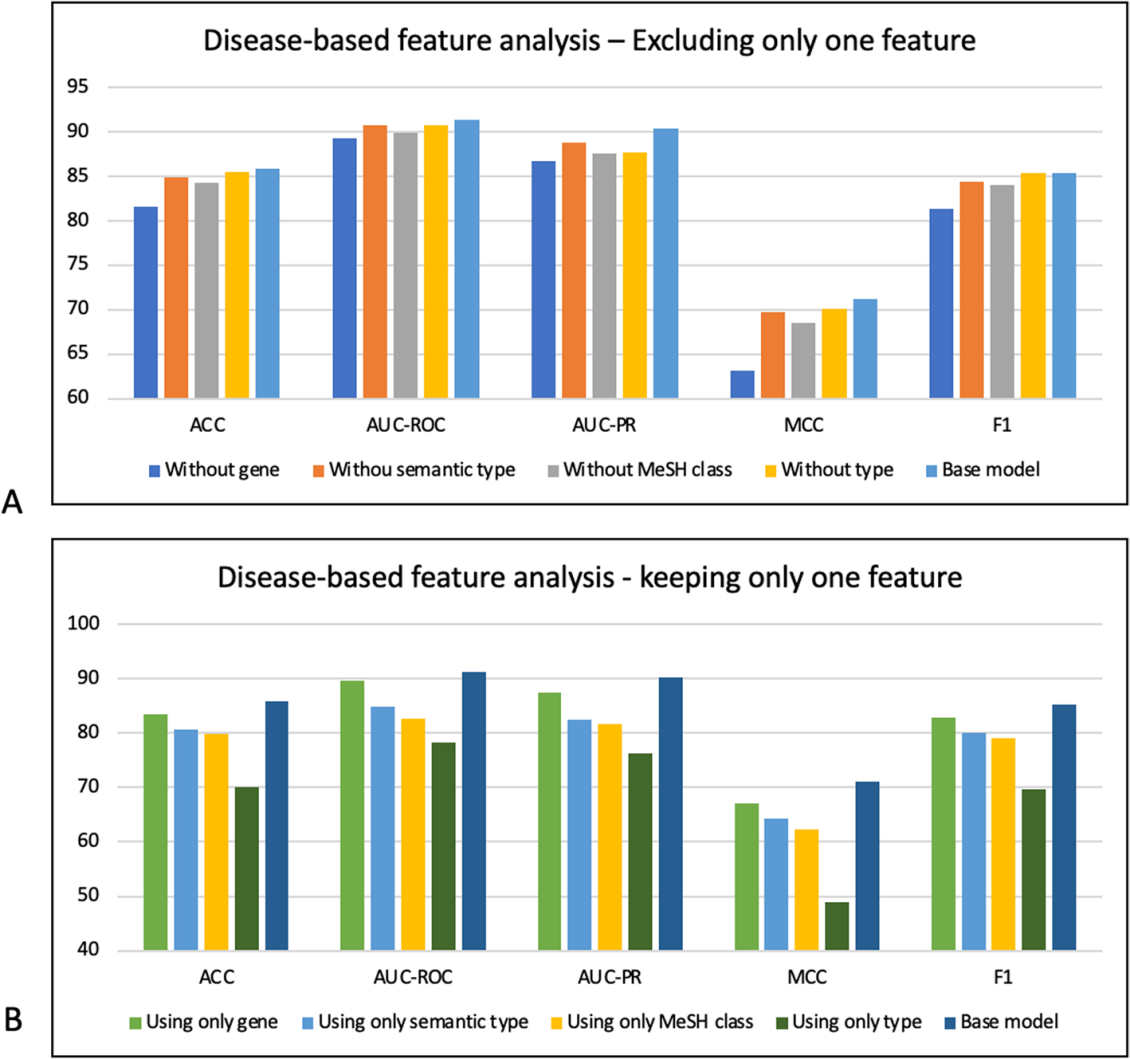
Disease-based feature analysis

When it comes to analyzing disease-based features, Fig. 6 illustrates the model's performance when a specific disease feature is omitted (A) or when only one disease feature is included (B). Our analysis also indicates that excluding the genes as the disease feature (as shown in Fig. 6-A, the dark blue bar) leads to a notable decrease in the base model’s performance (light blue bar). For instance, removing genes results in a 4% decrease in the ACC and an 8% drop in MCC for the base model. Conversely, using only genes as the disease feature (as shown in Fig. 6-B, the light green bar), results in a milder decline in the base model’s performance (the dark blue bar), specifically, a 2% decrease in ACC and a 4% decrease in MCC. In contrast, substituting genes with other features causes more substantial changes, approximately a 7% decrease in ACC and a 10% decrease in MCC.

These results propose genes as a crucial feature for diseases. The other studies also show the important role of genes in the development of various diseases and their importance in facing DR problem [54, 55]. Moreover, biological studies reveal that analyzing genes can significantly enhance the identification of disease-causing pathogens [22].

In conclusion, our analysis reveals that although employing the side effect as the drug feature and genes as the disease feature can significantly impact the model's performance, incorporating all features is recommended for obtaining better results.

### Comparison of DrugRep-HeSiaGraph with previous methods

In order to compare the effectiveness of DrugRep-HeSiaGraph, we use DRP-VEM as an ensemble learning and FB model [16], DisDrugPred as a matrix factorization-based and HNB model [9], and DrugRep-KG [22], as a knowledge graph-based model in two ways: the input representation and the model performance. Since the considered feature sets between the models are the same, they are comparable. However, as DisDrugPred is a regression model and DRP-VEM, DrugRep-KG and DrugRep-HeSiaGraph are classification models, only AUC-ROC and AUC-PR criteria are reported for DisDrugPred model.

#### Input representation

As discussed earlier, finding a proper drug and disease representation is a main challenge in the DR problem and various studies have employed different strategies to tackle this issue. In the context of our comparison, DisDrugPred is excluded, which is regression model utilizing a matrix factorization approach. For this purpose, three experiments are conducted based on each model representation, applying the same training and test sets as below:

- Binary representation employed by DRP-VEM model
- Word2Vec representation employed by DrugRep-KG model
- HeSiaNet-based representation employed by DrugRep-HeSiaGraph model

The details of each experiment are provided below. In order to ensure a fair comparison, we utilize the same training and test sets for all models. Additionally, the features of all models are derived from DDKG-V1, ensuring consistent input data across the experiments. This approach allows us to directly compare the performance of each model while controlling for any potential variations in the dataset or feature selection.

**Fig. 7:**
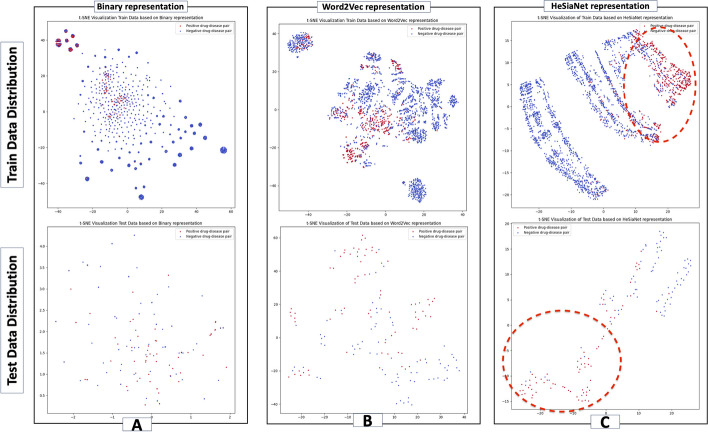
Comparison of models based on drug-disease pairs representation. The upper and bottom row demonstrate the train and test data distribution in the space, respectively. The column A represent the Binary representation, the column B shows the Word2Vec representation, and the column C illustrates the HeSiaNet representation

*Binary representation employed by DRP-VEM model* In this study, a binary vector is generated based on the considered features for drug and disease representations. Each feature is assigned a position in the vector, where a value of 1 indicates a relationship between the drug or disease and the corresponding feature, and 0 shows no relationship. Then, the drug-disease pairs are fed to the model based on the concatenation of drug’s and disease’s binary vectors. The length of this vector is equal to the summation of all feature items (13,869). To evaluate the effectiveness of this representation, its distribution is visualized using t-SNE in a 2D space. Figure 7-A illustrates the t-SNE plots of the training and test data. Positive instances, representing associated drug-disease pairs, are depicted in red, while negative instances are shown in blue. Notably, it demonstrates no clear separation between positive and negative drug-disease pairs. According to the plots, it becomes apparent that the positive and negative data points are not distinguishable, indicating that the binary representation alone is not a reliable representation for classification purposes.

*Word2Vec representation employed by DrugRep-KG model* As aforementioned, the DrugRep-KG model employs Word2Vec to generate embeddings for drugs and diseases. For a given drug-disease pair, the corresponding vectors are extracted and concatenated to feed the model. To evaluate the effectiveness of this representation, the drug-disease pairs of training and test sets are visualized by t-SNE. The resulting t-SNE plots are presented in Fig. 7-B for training and test sets.

Upon analyzing Fig. 7-B for training data, it clearly shows that the distribution of positive and negative pairs is relatively better separated compared to the binary representation experiment. However, the separation is not significantly distinct, indicating that there is still room for improvement in effectively distinguishing between positive and negative pairs.

Similarly, although Fig. 7-B for test data displays some degree of separation between positive and negative pairs, it is not as pronounced as desired, demonstrating that the embeddings alone may not provide a sufficiently discriminative representation for accurate classification.

*HeSiaNet-based representation employed by DrugRep-HeSiaGraph* In our proposed method, the main objective is to enhance the representation of drugs and diseases. To achieve this, a heterogeneous siamese neural network called HeSiaNet is introduced that takes the extracted Word2Vec embeddings of drugs and diseases as inputs and passes them through two distinct channels denoted as $f\left( {{\mathbb{E}}_{r} } \right)$ and $g\left( {{\mathbb{E}}_{p} } \right)$, respectively. These channels of HeSiaNet embed drugs and diseases in the latent space. After that, the output of these networks is subtracted ($\varepsilon_{h} = \left| {\varepsilon_{r} - \varepsilon_{p} } \right|$) in order to bring associated drugs and diseases closer in the unified latent space and then go through a hidden layer for predicting whether a given drug-disease pair is associated or not. By employing this approach, HeSiaNet leverages the power of SNN models to improve the representation of drugs and diseases.

To evaluate the effectiveness of this approach, we feed t-SNE with ${\upvarepsilon }_{h} = \left| {{\upvarepsilon }_{r} - {\upvarepsilon }_{p} } \right|$ as the embedded representation of drug-disease pairs in the unified latent space to visualize the training and test sets, illustrated in Fig. 7-C.

Upon analyzing Fig. 7-C, we observe that the train data is indeed better separated compared to the previous experiments, and the positive and negative instances are more distinguishable. Also, the test set shows better separation. The distribution of the data points is more well-organized in the lower-dimensional space, suggesting a clearer structure and potentially indicating the presence of meaningful clusters. It indicates that the proposed DrugRep-HeSiaGraph model provides a more effective representation of drugs and diseases.

These results demonstrate the effectiveness of utilizing the DrugRep-HeSiaGraph input in improving the separation and organization of the train and test data based on the t-SNE visualization. This suggests that the proposed model may offer enhanced capabilities for the accurate representation of drug-disease pairs.

#### The prediction’s performance

In this subsection, we define four different versions of DDKG for the assessment of incorporating new relationships. This experimental setup will allow us to determine which knowledge graph perform better in capturing and utilizing drug-disease associations. The four versions of DDKG are defined as follows:

- DDKG-V1: The version is introduced in the section ‘Drug-disease knowledge graph step’, and includes all defined intra- and inter-relationship types. The primary goal of DDKG-V1 is to assess the effectiveness of considering intra- and inter-relationships in improving the accuracy of models.
- DDKG-V2: This version considers relationship types 1.1–1.5, 2.1–2.4, and 3.1, which are used by DrugRep-KG's DDKG and serves as a baseline for comparison. The purpose of this version is to assess the classification performance of the HeSiaNet method compared to the regression classifier employed by DrugRep-KG.
- DDKG-V3: This version defines relationship types 1.6–1.7 and 2.5 in addition to DDKG-V2 as the new intra-relationship types. The aim is to investigate the impact of intra-relationship types between drugs and diseases on model performance compared to DDKG-V2.
- DDKG-V4: This version incorporates the new inter-relationship type 3.2 in addition to the relationship types included in DDKG-V2. The goal is to determine the effect of inter-relationship types between drugs and diseases on the performance of the models in comparison to DDKG-V2.

Table 5 illustrates a comparison between the proposed model, DrugRep-KG, DisDrugPred, and DRP-VEM. The "Model" column shows the employed model, and the "DDKG Version" column indicates the applied DDKG version, ranging from DDKG-V1 to DDKG-V4. The subsequent columns present evaluation criteria scores for each model based on every version of DDKG. It is important to note that the evaluation is based on the same test set, and since DisDrugPred and DRP-VEM are not a KG method, the differences in DDKG versions are not important for them. We interpret the results in two ways: the effectiveness of DDKGs and the performance of the DrugRep-HeSiaGraph against other models.

**Table 5:** The comparison between the performance of DrugRep-HeSiaGraph and DrugRep-KG based on four versions of DDKGs

Model	DDKG version	ACC (%)	AUC-ROC (%)	AUC-PR (%)	BS (%)	MCC (%)	F1-score (%)
DrugRep-HeSiaGraph	DDKG-V1	$84.63 \pm 0.02$	$91.16 \pm 0.04$	$90.32 \pm 0.13$	$11.90 \pm 0.04$	$69.31 \pm 0.82$	$83.13 \pm 0.12$
	DDKG-V2	$83.44 \pm 0.10$	$90.63 \pm 0.07$	$90.09 \pm 0.19$	$12.01 \pm 0.05$	$67.53 \pm 0.13$	$80.54 \pm 0.27$
	DDKG-V3	$84.51 \pm 0.04$	$90.97 \pm 0.03$	$90.27 \pm 0.41$	$12.00 \pm 0.04$	$69.12 \pm 0.14$	$82.84 \pm 0.08$
	DDKG-V4	$83.91 \pm 0.08$	$91.15 \pm 0.10$	$90.17 \pm 0.27$	$12.06 \pm 0.03$	$67.90 \pm 0.35$	$82.08 \pm 0.19$
DrugRep-KG	DDKG-V1	$83.9$ 3	$91.00$	$90.30$	$11.97$	$67.90$	$82.83$
	DDKG-V2	$83.92$	$90.57$	$89.94$	$12.18$	$67.50$	$83.04$
	DDKG-V3	$82.45$	$91.03$	$90.18$	$12.15$	$64.99$	$82.73$
	DDKG-V4	$82.45$	$90.94$	$89.97$	$12.24$	$64.92$	$81.89$
DisDrugPred	–	–	58.73	53.45	38.91	–	–
DRP-VEM	–	52.33	55.37	55.12	29.76	32.43	52.35

The experiments demonstrate that the inclusion of new intra- and inter-relationship types in DDKG-V1 outperforms all other versions, including DDKG-V2, which serves as the base DDKG for both knowledge graph-based models. Notably, DDKG-V1 also improves the performance of DrugRep-KG as the base model compared to using DDKG-V2 in all metrics. Additionally, applying DDKG-V3 and DDKG-V4 enhances the performance of our model compared to using DDKG-V2 as the base DDKG. For instance, our model using DDKG-V3 achieves approximately 1.2% and 2% better results on ACC and MCC compared to when using DDKG-V2. Furthermore, since DDKG-V3 contains more information than DDKG-V4, the performance of both models using DDKG-V3 is better than applying DDKG-V4. Moreover, DrugRep-KG shows improved performance in specific criteria when DDKG-V3 and DDKG-V4 are used instead of its primary knowledge graph, DDKG-V2. These results demonstrate that adding new relationship types improves the performance of the model and DDKG-V1 is a better DDKG for exploring the associations between drugs and diseases in comparison to DDKG-V2.

**Fig. 8:**
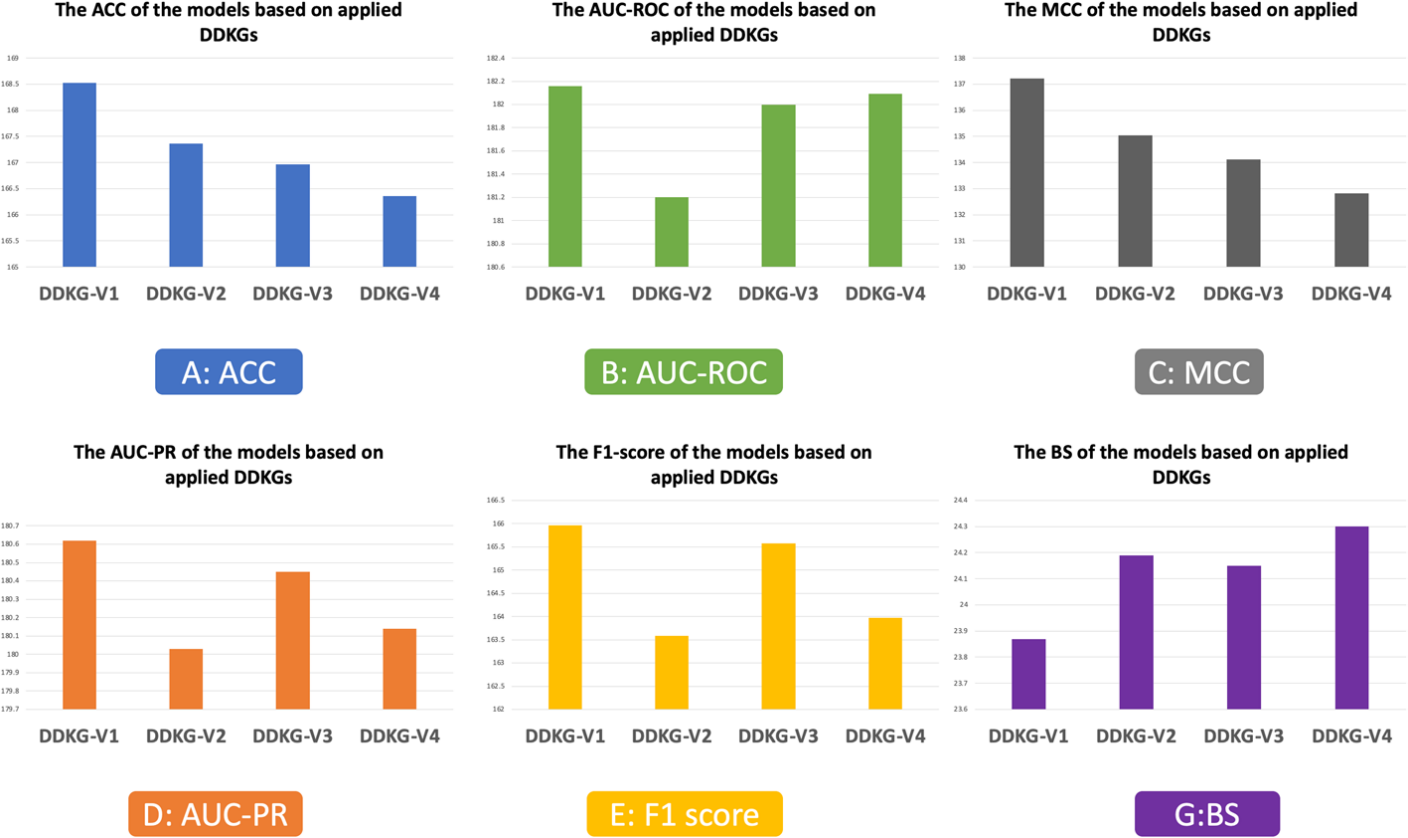
Comparison of the versions of DDKGs in performance of the models, based on each evaluation criterion, such as **A** ACC, **B** AUC-ROC, **C** MCC, **D** AUC-PR, **E** F1-score, and **G** BS

Figure 8 illustrates how different versions of DDKG affect the performance of the models across various criteria. The bar plots illustrate the overall performance of our proposed model and DrugRep-KG on each criterion across different versions of DDKG. The figure clearly shows that DDKG-V1 is more effective than the other versions, specifically DDKG-V2 as the baseline DDKG, while DDKG-V3 outperforms DDKG-V4. These results suggest that the choice of DDKG version can significantly impact on the performance of the knowledge graph-based models and highlight the importance of careful selection of relationship types in drug repurposing.

In the comparison of model’s performance viewpoint, our model outperforms three others on all metrics using DDKGs of different versions, and DisDrugPred predicts the associations poorly, as shown in Table 5. This demonstrates the effectiveness of incorporating heterogeneous channels in the HeSiaNet model and highlights the effectiveness of using neural networks in comparison to probabilistic models. Moreover, it shows KGs capture the data better than matrix factorization methods. Overall, the proposed model, with the addition of new relationships and the HeSiaNet, shows promise in improving the accuracy and effectiveness of predicting drug-disease associations.

### Comparison of homogeneous versus heterogeneous siamese neural network

The study hypothesizes that DrugRep-KG [22] may not optimally learn the unified latent space for drugs and diseases due to their inherent differences in nature. To address this issue, we suggest using a heterogeneous SNN model, HeSiaNet. The model is designed to handle the heterogeneity of drugs and diseases by allowing for varied neural architectures to be employed. However, if drugs and diseases are already in an optimal latent space, where they can be accurately represented, a homogeneous SNN (Hom-SNN) model may perform accurately without the need for a more complex model like HeSiaNet. In the following, we conduct an investigation using a Hom-SNN model and evaluate its effectiveness in comparison to HeSiaNet to test this hypothesis.

Homogeneous and heterogeneous SNNs are two deep learning models utilized for similarity analysis and comparison of input data using two channels. The main distinction between them is that Hom-SNN employs two parallel and identical channels with the same weights to process two input data points of the same type, whereas heterogeneous-SNN uses two different neural networks optimized for each input data type. As a result, even though they have a similar architecture, the weights of each channel are updated independently. Hom-SNNs are commonly used for tasks in which both input data come from the same source, while heterogeneous-SNNs are more suitable for tasks that involve input data of different types. Both types of networks are powerful tools for analyzing and comparing input data, and they can be used in a variety of applications. HeSiaNet is based on heterogeneous SNN models because it uses different types of data, including drugs and diseases.

To validate the hypothesis, we test the performance of DrugRep-HeSiaGraph by comparing Hom-SNN and HeSiaNet. The architecture of the applied Hom-SNN is the same as that of HeSiaNet, while the drug channel and disease channel share the weights, whereas in HeSiaNet, they are optimized independently. According to Table 6, the performance of HeSiaNet is significantly better than Hom-SNN. For instance, HeSiaNet outperforms Hom-SNN by approximately 3%, 6%, and 4% on AUC-ROC, AUC-PR, and MCC, respectively. This supports our assumption that DrugRep-KG cannot completely overcome the gap between the latent spaces and that applying HeSiaNet based on a heterogeneous network is the correct approach.

**Table 6:** The comparison of HeSiaNet as the heterogeneous-SNN and Hom-SNN as the homogeneous-SNN models

Model	AUC-ROC (%)	AUC-PR (%)	ACC (%)	BS (%)	MCC (%)	F1-score (%)
HeSiaNet	$84.2 \pm 0.29$	$91.08 \pm 0.04$	$90.35 \pm 0.13$	$11.99 \pm 0.05$	$68.51 \pm 1.22$	$82.31 \pm 0.03$
Hom- SNN	$81.56 \pm 0.34$	$85.68 \pm 0.91$	$81.58 \pm 0.30$	$12.48 \pm 0.83$	$64.38 \pm 2.25$	$78.46 \pm 1.09$

### Case study

The emergence of COVID-19 in late 2019, coupled with the urgent need for effective medications, has underscored the importance of drug repositioning. According to the World Health Organization (WHO), as of May 24th, 2023, the global confirmed cases of COVID-19 have surpassed over 699 million, with a staggering death toll of 6 million [56]. In the initial stages of the pandemic, healthcare professionals were compelled to rely on existing medications to manage the virus while awaiting the development of novel treatments. Consequently, extensive research has been conducted to explore the application of drug repositioning techniques to combat COVID-19. This strategy involves repurposing approved drugs for other diseases to address the novel coronavirus. Thus, the objective of this section is to identify effective treatments for COVID-19. In this context, we evaluate the performance of DrugRep-HeSiaGraph in identifying potential therapeutic options for COVID-19 as a new disease scenario to check the generalization power of the model.

In the subsequent analysis, we assess the efficacy of the proposed model in three distinct aspects:

- *Assessment of potential treatments* The proposed model’s ability to find appropriate treatments for COVID-19 is assessed by using the suggested potential treatments of the DrugBank database,
- *Assessment of model’s suggested drugs* The top 10 suggested drugs of the proposed model for the treatment of COVID-19 are examined using literature searches,
- *Assessment of DPP-4 inhibitors* The performance of dipeptidyl peptidase 4 (DPP-4) inhibitors as prospective drug candidates for COVID-19, which model predicts a high probability for the therapeutic association between these and COVID-19, are examined. This investigation is rooted in the hypothesis that DPP-4 could serve as a primary receptor for the SARS-CoV-2 virus. By utilizing the DrugRep-HeSiaGraph model, we examine the effectiveness of DPP-4 inhibitors in combating COVID-19, shedding light on their potential therapeutic value in mitigating viral infection.

#### Assessment of potential treatment for COVID-19

Our investigation utilizes the DrugBank database [35], which includes a COVID-19 section dedicated to information on 74 experimental and unapproved treatments for COVID-19. The set of drugs we've selected $\left( \varphi \right)$ includes seven medications that DrugBank has suggested [35]: dexamethasone (DB01234), methylprednisolone (DB00959), chloroquine (DB00608), colchicine (DB01394), azithromycin (DB00207), ibuprofen (DB01050), and fingolimod (DB08868). It is important to note that the therapeutic association between these drugs and COVID-19, the targeted disease, has not received official approval. Nevertheless, our model successfully predicts the potential efficacy of these drugs against COVID-19. The corresponding probability of associations between COVID-19 and the aforementioned drugs is detailed in Table 7. These findings suggest that DrugRep-HeSiaGraph holds promise as a valuable tool for identifying potential treatments to combat COVID-19 by repurposing existing drugs.

**Table 7:** The probability of association between COVID-19 and drugs in DrugBank

Drug name	DrugRep-HeSiaGraph probability (%)	DrugRep-KG probability (%)
Dexamethasone (DB01234)	97.79	92.50
Methylprednisolone (DB00959)	97.61	90.80
Chloroquine (DB000608)	96.57	87.50
Colchicine (DB01394)	96.77	87.00
Azithromycin (DB00207)	90.87	73.70
Ibuprofen (DB01050)	86.19	62.30
Fingolimod (DB08868)	94.41	62.21

#### Assessment the model’s suggested drugs for COVID-19

In order to showcase the effectiveness of the model in dealing with real-life problems and to assess the reliability of suggested associations, we train the DrugRep-HeSiaGraph using all associations in the dataset. It is important to note that there is no therapeutical association between COVID-19 and the drugs. We then feed the pairs of COVID-19 and all drugs in the drug set to the model to predict their associations. Since the model assigns a probability score to each association, the pairs are sorted based on decreasing probability, and the top 10 drugs suggested by the model are listed in Table 8. Furthermore, we compare the model's performance to that of DrugRep-KG and DisDrugPred, repeating the experiment with these models. The results of all three of them are provided in Table 8 for evaluation purposes.

**Table 8:** The Top 10 drug suggestions of DrugRep-HeSiaGraph against COVID-19, and the DrugRep-KG and DisDrugPred predictions

Drug name	DrugRep- HeSiaGraph	DrugRep-KG	DisDrugPred	Evidence
Fusidic acid (DB02703)	96.47	85.89	56.12	[57]
Fluocinonide (DB01047)	96.42	93.12	48.76	[58, 59]
Alclometasone (DB00240)	96.37	94.23	49.66	[60, 61]
Erythromycin (DB00199)	96.35	74.55	59.14	[62, 63]
Spironolactone (DB00421)	96.33	91.67	39.33	[64, 65]
Prednisolone (DB00860)	96.31	86.31	68.74	[66]
Ergocalciferol (DB00153)	96.29	90.12	22.46	[67, 68]
Medrysone (DB00253)	96.29	89.34	70.12	[69]
Fludrocortisone (DB00687)	96.29	91.76	45.45	[70, 71]
Flurandrenolide (DB00846)	96.27	90.38	34.65	–

According to this table, of the first 10 suggested drugs, nine are evidenced in other studies. Moreover, although DrugRep-KG also has similar predictions, the probability of these associations is estimated to be lower than our model. DisDrugPred predicts 4 of these 10 associations, which shows the poor performance of this model in finding potential treatments for novel diseases.

#### Assessment of DPP-4 inhibitors for COVID-19

In the continuation of the previous section and investigating more potential treatments for COVID-19, we find an association with high probability between gliptins and COVID-19 as a diabetic medicines. Diabetes mellitus encompasses a range of metabolic disorders that result in chronically elevated blood sugar levels due to issues related to insulin secretion, insulin action, or both [72]. The effects of different drug types used to treat diabetes on the outcomes of individuals infected with COVID-19 have been a subject of debate. Researchers have specifically focused on investigating the potential of dipeptidyl peptidase 4 (DPP-4) inhibitors [73], as emerging evidence suggests that the DPP-4 enzyme may play a role in the development of COVID-19 disease [74].

The DPP-4 enzyme plays a crucial role in regulating glucose levels by increasing insulin secretion and reducing glucagon secretion [75]. Moreover, DPP-4 is known as the main receptor for the Middle East respiratory syndrome coronavirus (MERS-CoV) [76]. Therefore, according to studies there is a chance, that the spike glycoprotein of SARS-CoV-2 can also bind with DPP-4 [77, 78]. In this section, our objective is to explore the potential of DPP-4 enzyme as the receptor of SARS-CoV-2 through molecular docking, and the role of DPP-4 inhibitors in treatment of COVID-19.

**Fig. 9:**
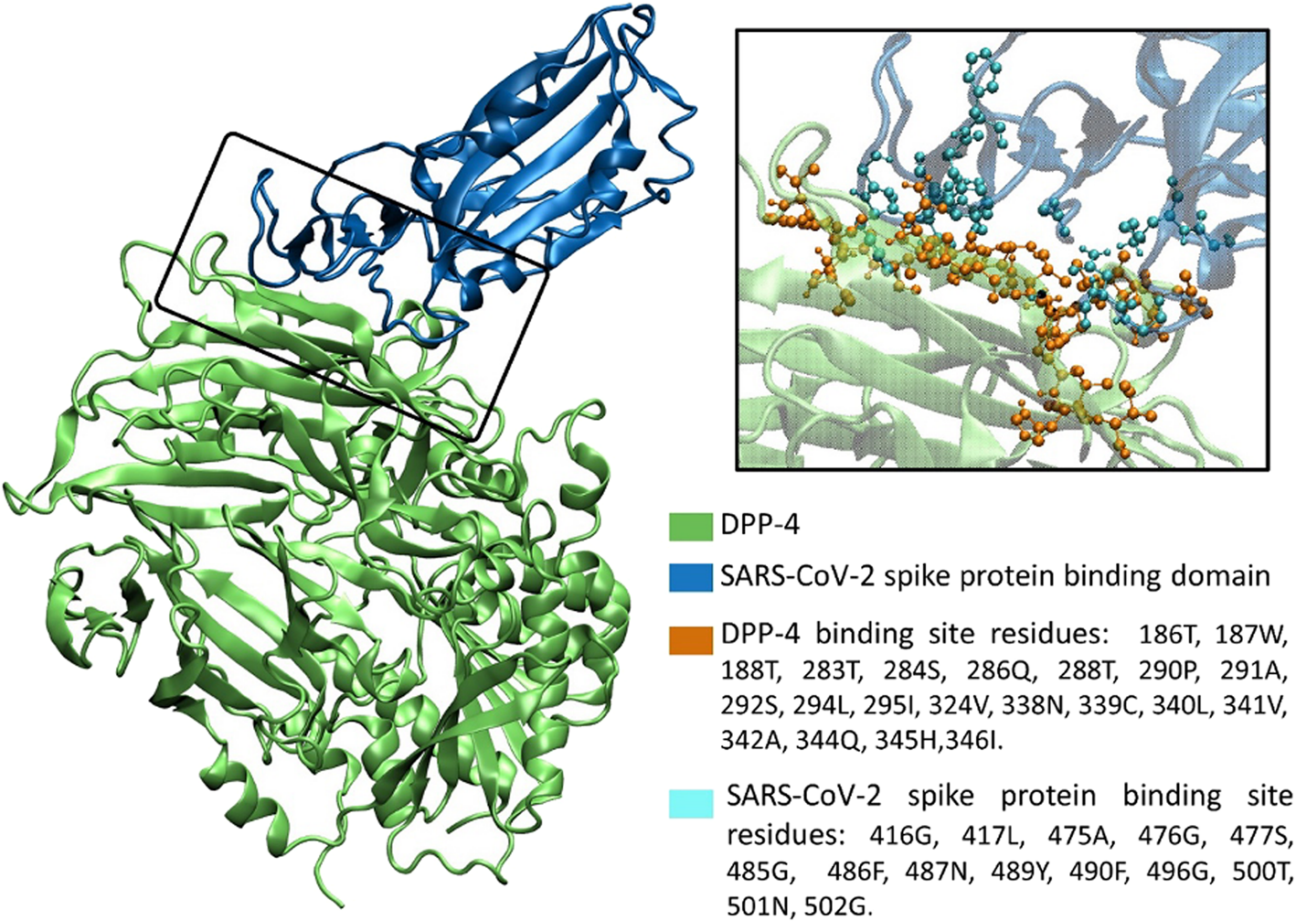
The complex structure of DPP-4 in interaction with the SARS-CoV-2 spike protein binding domain

To assess how SARS-CoV-2 spike protein and DPP-4 interact, the 3D structures of these proteins are obtained from the Protein Data Bank [79]. Specifically, the receptor-binding domain of the spike protein and DPP-4 are extracted from two complexes: the SARS-CoV-2 spike receptor-binding domain bound with Angiotensin-converting enzyme 2 (ACE2) protein (PDB ID: 6M0J), and the MERS-CoV spike receptor-binding domain complexed with DPP4 (PDB ID: 4L72), respectively. The Reduce program [80] is utilized to add hydrogen atoms to the 3D structures. Then, the SARS-CoV-2 spike protein and DPP-4 are docked using the HADDOCK 2.4 web server [81, 82] which employs an information-driven flexible docking approach. The active residues are determined based on the PDB complex structures, while default values are used for other HADDOCK settings. The top cluster of HADDOCK results contains the most reliable conformations according to the averaged HADDOCK scores which are calculated by combining various energies and buried surface area into a linear combination. Figure 9 displays the binding site residues and optimal complex structure of SARS-CoV-2 spike protein with DPP-4, selected from the top cluster of HADDOCK results with a HADDOCK score of -125.5 ± 2.1. Moreover, VMD 1.9.3 [83] is used to show the complex structure and performs binding site analysis. The HADDOCK score and complex structure confirm the role of DPP-4 as a receptor for the SARS-CoV-2 spike protein.

DPP-4 inhibitors (DPP-4Is), also known as gliptins, are a class of medications used to treat type 2 diabetes. It is shown that gliptins may have a potential role in decreasing the severity of a SARS-CoV-2 infection [84] and also in counteracting the impact of the virus. Moreover, DPP-4Is have been found to suppress T cell proliferation, leading to a decrease in the production of pro-inflammatory cytokines. As a result, the occurrence of a cytokine storm may be reduced. This effect has been observed in studies where patients taking DPP-4Is have exhibited a 30% lower incidence of autoimmune diseases [85].

The most well-known approved gliptins include sitagliptin (DB01261), vildagliptin (DB04876), linagliptin (DB08882), saxagliptin (DB06335), and alogliptin (DB06203). These drugs are also part of our applied drug set ($\varphi$). These drugs have demonstrated they have the potential to treat COVID-19.

DPP-4Is can be effective TLR4 antagonists for COVID-19 patients who experience a cytokine storm, as DPP-4 is expressed in immune cells that contribute to COVID-19 immunopathology [86]. Research has shown that alogliptin can effectively inhibit TLR4-mediated ERK activation and MMP-1 expression by monocytes [87]. Conversely, sitagliptin has been identified as a TLR4 activation inhibitor, which may be beneficial in reducing the interaction between SARS-CoV-2 and TLR4, leading to decreased inflammation. Sitagliptin has the ability to selectively reduce proinflammatory cytokine levels in COVID-19 patients by blocking NF-κB signaling [88]. Furthermore, sitagliptin, alogliptin, vildagliptin, saxagliptin, and linagliptin have all demonstrated potential in mitigating the immunopathogenesis, cytokine storm, and organ damage that result from SARS-CoV-2 infection [89].

**Fig. 10:**
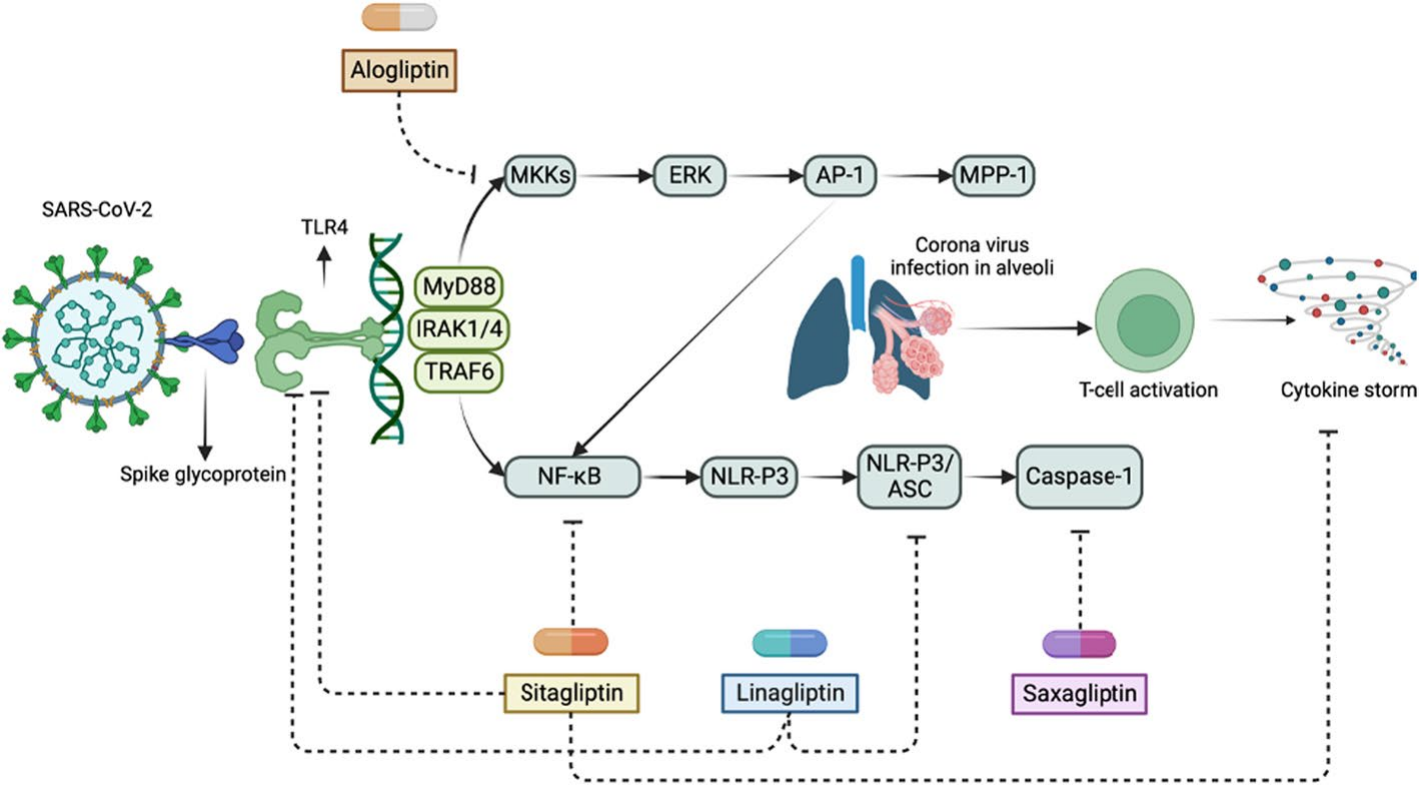
An overview of DPP-4 inhibitors

DPP-4Is also have anti-inflammatory properties, which could be useful for mitigating post-COVID cardiac inflammation and heart failure. Notably, cardiac hyperinflammation is a significant cause of death in COVID-19 patients, with TLR4 and NLRP3 activation identified as major immunopathological hallmarks. Blockage of the NLRP3/ASC inflammasome is a known mechanism used by linagliptin to improve heart inflammation and dysfunction [90]. Additionally, linagliptin has been shown to inhibit TLR4 activation, which suggests that it may also have the potential to reduce cardiac inflammation in COVID-19 patients [91]. In addition, hypertension is a frequently occurring comorbidity in COVID-19 patients, and studies have shown that saxagliptin, vildagliptin, and sitagliptin have the potential to reduce both systolic and diastolic blood pressure. This effect may be beneficial in managing the blood pressure of COVID-19 patients and lowering the risk of ICU admission, heart failure, and mortality [92]. Figure 10 provides an overview of the molecular targets of various DPP-4 inhibitors that can help alleviate the immunopathological effects of SARS-CoV-2. These targets are linked to TLR4 and the associated signaling pathways.

So, DPP-4 can interact with the SARS-CoV-2 spike glycoprotein and exacerbate inflammatory conditions in various organs, leading to fatal consequences. Repurposing DPP-4Is for treating SARS-CoV-2 infection may prevent inflammation by inhibiting DPP-4 or TLR4 activation. In addition, DPP-4Is can improve diabetogenic conditions and reduce blood pressure, which are common comorbidities in COVID-19 patients. These factors make DPP-4Is a promising treatment option for COVID-19, particularly in diabetic patients.

Table 9 presents the probabilities of an association between DPP-4Is and COVID-19, as predicted by DrugRep-HeSiaGraph. These probabilities provide further evidence that DPP-4Is may be effective in the treatment of COVID-19.

**Table 9:** The predictions of DrugRep-HeSiaGraph in identifying the association between DPP-4Is and COVID-19

Drug name	Model prediction (%)
Sitagliptin	70.25
Vildagliptin	92.29
Linagliptin	89.60
Saxagliptin	91.17
Alogliptin	93.30

According to conducted experiments, the DrugRep-HeSiaGraph can effectively be utilized in suggesting treatments for new diseases.

## Conclusion

In conclusion, this paper introduced the DrugRep-HeSiaGraph method, which improved the performance of DrugRep-KsG as a baseline model. It extended the DDKG by defining a new intra- and inter- relationships between drugs and diseases features. Additionally, the DrugRep-HeSiaGraph model suggested using a heterogeneous siamese neural network for enriching the embedding of drugs and diseases as well as making an accurate prediction for potential drug-disease associations. Moreover, this study highlighted the importance of repurposing existing medications for the treatment of COVID-19 and the potential benefits of DPP-4Is like Saxagliptin. However, further clinical trials are necessary to confirm its effectiveness and safety in treating COVID-19. It is important to note that drug repurposing studies should always be conducted within a rigorous scientific framework to ensure the safety and efficacy of any proposed treatments.

However, we aim to improve the model by exploring new representations instead of the current knowledge graph embedding techniques. It is important to note that the current knowledge graph embedding model is dependent on the trained entities and may not be generalized to predict new diseases or drugs. We believe that future research in this area will lead to even more effective drug repositioning strategies, ultimately improving patient outcomes.

### Supplementary Information

**Additional file 1: Table S1.** Provide the corresponding evaluation scores of using different combination of drug features, as well as disease features. According to our analysis using Side effect effect as a drug feature and Gene as a disease feature is crucial in facing DR problem.

## Data Availability

The code and data for DrugRep-HeSiaGraph are publicly available on https://github.com/CBRC-lab/DrugRep-HeSiaGraph.

## Abbreviations

DR: Drug repurposing
FB: Feature-based
HNB: Heterogenous network-based
KG: Knowledge graph
DDKG: Drug-disease knowledge graph
SNN: Siamese neural network
ATC: Anatomical therapeutic chemical
UMLS: Unified medical language system
CBOW: Continuous bag of words
Hom-SNN: Homogeneous SNN
AUC-ROC: Area under curve-receiver operating characteristic
AUC-PR: Area under curve-precision recall
ACC: Accuracy
BS: Berier score
MCC: Matthew’s correlation coefficient
DPP-4: Dipeptidyl peptidase-4
Mers-CoV: Middle east respiratory syndrome coronavirus
ACE2: Angiotensin-converting enzyme 2
DPP-4I: DPP-4 inhibitor

## References

[1] Zhou S, Wang F, Hsieh T-C, Wu JM, Wu E (2013). Thalidomide–a notorious sedative to a wonder anticancer drug. Curr Med Chem.

[2] Johnson NP (2014). Metformin use in women with polycystic ovary syndrome. Ann Transl Med.

[3] Moridi M, Ghadirinia M, Sharifi-Zarchi A, Zare-Mirakabad F (2019). The assessment of efficient representation of drug features using deep learning for drug repositioning. BMC Bioinform.

[4] Chen H, Zhang Z, Zhang J (2021). In silico drug repositioning based on the integration of chemical, genomic and pharmacological spaces. BMC Bioinform.

[5] Shao M, Jiang L, Meng Z, Xu J (2022). Computational drug repurposing based on a recommendation system and drug–drug functional pathway similarity. Molecules.

[6] Abbasi K, Razzaghi P, Poso A, Ghanbari-Ara S, Masoudi-Nejad A (2021). Deep learning in drug target interaction prediction: current and future perspectives. Curr Med Chem.

[7] Masoudi-Sobhanzadeh Y, Omidi Y, Amanlou M, Masoudi-Nejad A (2019). Trader as a new optimization algorithm predicts drug-target interactions efficiently. Sci Rep.

[8] Masoudi-Sobhanzadeh Y, Omidi Y, Amanlou M, Masoudi-Nejad A (2019). DrugR+: a comprehensive relational database for drug repurposing, combination therapy, and replacement therapy. Comput Biol Med.

[9] Xuan P, Cao Y, Zhang T, Wang X, Pan S, Shen T (2019). Drug repositioning through integration of prior knowledge and projections of drugs and diseases. Bioinformatics.

[10] Zeng X, Zhu S, Liu X, Zhou Y, Nussinov R, Cheng F (2019). DeepDR: a network-based deep learning approach to in silico drug repositioning. Bioinformatics.

[11] Luo H, Wang J, Li M, Luo J, Ni P, Zhao K (2019). Computational drug repositioning with random walk on a heterogeneous network. IEEE/ACM Trans Comput Biol Bioinform.

[12] Meng Y, Lu C, Jin M, Xu J, Zeng X, Yang J (2022). A weighted bilinear neural collaborative filtering approach for drug repositioning. Br Bioinform.

[13] Pan X, Yun J, Coban Akdemir ZH, Jiang X, Wu E, Huang JH (2023). AI-DrugNet: a network-based deep learning model for drug repurposing and combination therapy in neurological disorders. Comput Struct Biotechnol J.

[14] Zhao BW, Su XR, Hu PW, Ma YP, Zhou X, Hu L (2022). A geometric deep learning framework for drug repositioning over heterogeneous information networks. Br Bioinform.

[15] Zhao BW, Wang L, Hu PW, Wong L, Su XR, Wang BQ (2023). Fusing higher and lower-order biological information for drug repositioning via graph representation learning. IEEE Trans Emerg Top Comput.

[16] Ghorbanali Z, Zare-Mirakabad F, Mohammadpour B. DRP-VEM: drug repositioning prediction using voting ensemble. 2021. 10.48550/arxiv.2110.01403

[17] Sang S, Yang Z, Liu X, Wang L, Lin H, Wang J (2019). GrEDeL: a knowledge graph embedding based method for drug discovery from biomedical literatures. IEEE Access.

[18] Nian Y, Hu X, Zhang R, Feng J, Du J, Li F (2022). Mining on Alzheimer’s diseases related knowledge graph to identity potential AD-related semantic triples for drug repurposing. BMC Bioinform.

[19] Sosa DN, Derry A, Guo M, Wei E, Brinton C, Altman RB (2020). A literature-based knowledge graph embedding method for identifying drug repurposing opportunities in rare diseases. Pac Symp Biocomput.

[20] Zhu Y, Che C, Jin B, Su C, Wang F (2020). Knowledge-driven drug repurposing using a comprehensive drug knowledge graph. Health Inform J.

[21] Kanatsoulis CI, Sidiropoulos ND (2021). Tex-graph: coupled tensor-matrix knowledge-graph embedding for COVID-19 drug repurposing. SIAM Int Conf Data Min SDM.

[22] Ghorbanali Z, Zare-Mirakabad F, Akbari M, Salehi N, Masoudi-Nejad A (2023). DrugRep-KG: toward learning a unified latent space for drug repurposing using knowledge graphs. J Chem Inf Model.

[23] Al-Saleem J, Granet R, Ramakrishnan S, Ciancetta NA, Saveson C, Gessner C (2021). Knowledge graph-based approaches to drug repurposing for COVID-19. J Chem Inf Model.

[24] Bordes A, Usunier N, Garcia-Duran A, Weston J, Yakhnenko O. Translating Embeddings for Modeling Multi-relational Data. Adv Neural Inf Process Syst. 2013;26.

[25] Lin Y, Liu Z, Sun M, Liu Y, Zhu X (2015). Learning entity and relation embeddings for knowledge graph completion. Proc Natl Conf Artif Intell.

[26] Wang Z, Zhang J, Feng J, Chen Z (2014). Knowledge graph embedding by translating on hyperplanes. Proc Natl Conf Artif Intell.

[27] Grover A, Leskovec J. Node2vec: scalable feature learning for networks. In: Proceedings of the ACM SIGKDD international conference on knowledge discovery and data mining. pp. 855–64. 10.1145/2939672.2939754 PMC5108654 27853626

[28] Trouillon T, Welbl J, Riedel S, Gaussier É, Bouchard G. Complex embeddings for simple link prediction; 2016.

[29] Di Gennaro G, Buonanno A, Palmieri FAN (2021). Considerations about learning Word2Vec. J Supercomput.

[30] Chicco D (2021). Siamese neural networks: an overview. Methods Mol Biol.

[31] Kim S, Chen J, Cheng T, Gindulyte A, He J, He S (2019). PubChem 2019 update: improved access to chemical data. Nucleic Acids Res.

[32] Saadat M, Behjati A, Zare-Mirakabad F, Gharaghani S. Drug-target binding affinity prediction using transformers. bioRxiv. 2022;2021.09.30.462610.

[33] Soleymani Babadi F, Razaghi-Moghadam Z, Zare-Mirakabad F, Nikoloski Z. Prediction of metabolite–protein interactions based on integration of machine learning and constraint-based modeling. Bioinformatics Advances. 2023;3. 10.1093/bioadv/vbad098 PMC10374491 37521309

[34] Besharatifard M, Ghorbanali Z, Zare-Mirakabad F (2023). Adverse drug reaction prediction using voting ensemble training approach. AUT J Math Comput.

[35] Wishart DS, Feunang YD, Guo AC, Lo EJ, Marcu A, Grant JR (2018). DrugBank 50: a major update to the DrugBank database for 2018. Nucleic Acids Res.

[36] Szklarczyk D, Gable AL, Lyon D, Junge A, Wyder S, Huerta-Cepas J (2019). STRING v11: protein-protein association networks with increased coverage, supporting functional discovery in genome-wide experimental datasets. Nucleic Acids Res.

[37] Bateman A, Martin MJ, Orchard S, Magrane M, Agivetova R, Ahmad S (2021). UniProt: The universal protein knowledgebase in 2021. Nucleic Acids Res.

[38] Kuhn M, Letunic I, Jensen LJ, Bork P (2016). The SIDER database of drugs and side effects. Nucleic Acids Res.

[39] Piñero J, Ramírez-Anguita JM, Saüch-Pitarch J, Ronzano F, Centeno E, Sanz F (2020). The DisGeNET knowledge platform for disease genomics: 2019 update. Nucleic Acids Res.

[40] Brown AS, Patel CJ (2017). A standard database for drug repositioning. Sci Data.

[41] Goodfellow I, Bengio Y, Courville A. Deep learning. MIT Press; 2016.

[42] Van Der Maaten L, Hinton G (2008). Visualizing data using t-SNE. J Mach Learn Res.

[43] Kumar R, Indrayan A (2011). Receiver operating characteristic (ROC) curve for medical researchers. Indian Pediatr.

[44] Sofaer HR, Hoeting JA, Jarnevich CS (2019). The area under the precision-recall curve as a performance metric for rare binary events. Methods Ecol Evol.

[45] Hernández-Orallo J, Flach PA, Ferri C. Brier curves: a New cost-based visualisation of classifier performance; 2011.

[46] Chicco D, Tötsch N, Jurman G (2021). The Matthews correlation coefficient (MCC) is more reliable than balanced accuracy, bookmaker informedness, and markedness in two-class confusion matrix evaluation. BioData Min.

[47] Rehurek R, Rehurek R, Sojka P. Software framework for topic modelling with large corpora. In: Proceedings of the LREC 2010 workshop on new challenges for NLP Frameworks. 2010;45–50.

[48] Chollet F, et al. Keras: Deep learning for humans. 2015. https://keras.io/. Accessed 14 May 2023.

[49] Lemaître G, Nogueira F, Aridas CK. Imbalanced-learn: a python toolbox to tackle the curse of imbalanced datasets in machine learning; 2016.

[50] Chawla NV, Bowyer KW, Hall LO, Kegelmeyer WP (2011). SMOTE: synthetic minority over-sampling technique. J Artif Intell Res.

[51] Bulinski A, Dimitrov D (2021). Statistical estimation of the Kullback-Leibler divergence. Mathematics.

[52] Zhang P, Wang F, Hu J, Sorrentino R (2013). Exploring the relationship between drug side-effects and therapeutic indications. AMIA Ann Symp Proc.

[53] Lakizadeh A, Hassan Mir-Ashrafi SM (2021). Drug repurposing improvement using a novel data integration framework based on the drug side effect. Inform Med Unlocked.

[54] Lee BKB, Tiong KH, Chang JK, Liew CS, Abdul Rahman ZA, Tan AC (2017). DeSigN: connecting gene expression with therapeutics for drug repurposing and development. BMC Genom.

[55] Donner Y, Kazmierczak S, Fortney K (2018). Drug repurposing using deep embeddings of gene expression profiles. Mol Pharm.

[56] WHO Coronavirus (COVID-19) Dashboard|WHO Coronavirus (COVID-19) Dashboard With Vaccination Data. https://covid19.who.int/. Accessed 30 May 2023.

[57] Hetmann M, Langner C, Durmaz V, Cespugli M, Köchl K, Krassnigg A (2023). Identification and validation of fusidic acid and flufenamic acid as inhibitors of SARS-CoV-2 replication using DrugSolver CavitomiX. Sci Rep.

[58] Alabdulaaly L, Sroussi H, Epstein JB (2022). New onset and exacerbation of oral lichenoid mucositis following SARS-CoV-2 infection or vaccination. Oral Dis.

[59] Ng AT, Miller A, Bodemer AA, Drolet BA, Arkin L (2022). Purple toes following critical COVID-19 infection. Pediatr Dermatol.

[60] Kuleshov MV, Stein DJ, Clarke DJB, Kropiwnicki E, Jagodnik KM, Bartal A (2020). The COVID-19 drug and gene set library. Patterns.

[61] Kouznetsova VL, Zhang A, Tatineni M, Miller MA, Tsigelny IF (2020). Potential COVID-19 papain-like protease PLpro inhibitors: repurposing FDA-approved drugs. PeerJ.

[62] Adebisi YA, Jimoh ND, Ogunkola IO, Uwizeyimana T, Olayemi AH, Ukor NA (2021). The use of antibiotics in COVID-19 management: a rapid review of national treatment guidelines in 10 African countries. Trop Med Health.

[63] Poddighe D, Aljofan M (2020). Clinical evidences on the antiviral properties of macrolide antibiotics in the COVID-19 era and beyond. Antivir Chem Chemother.

[64] Cousins HC, Altman RB (2023). Association between spironolactone use and COVID-19 outcomes in population-scale claims data: a retrospective cohort study. medRxiv.

[65] Cadegiani FA, Goren A, Wambier CG (2020). Spironolactone may provide protection from SARS-CoV-2: targeting androgens, angiotensin converting enzyme 2 (ACE2), and renin-angiotensin-aldosterone system (RAAS). Med Hypotheses.

[66] Ghanei M, Solaymani-Dodaran M, Qazvini A, Ghazale AH, Setarehdan SA, Saadat SH (2021). The efficacy of corticosteroids therapy in patients with moderate to severe SARS-CoV-2 infection: a multicenter, randomized, open-label trial. Respir Res.

[67] Ohaegbulam KC, Swalih M, Patel P, Smith MA, Perrin R (2020). Vitamin D supplementation in COVID-19 patients: a clinical case series. Am J Ther.

[68] Zhang Y, Li J, Yang M, Wang Q (2023). Effect of vitamin D supplementation on COVID-19 patients: a systematic review and meta-analysis. Front Nutr.

[69] Mirmohammadi SM, Kianmehr A, Sabbaghian A, Mohebbi A, Shahbazmohammadi H, Sheykharabi M (2022). In silico drug repurposing against SARS-CoV-2 using an integrative transcriptomic profiling approach: Hydrocortisone and Benzhydrocodone as potential drug candidates against COVID-19. Infect Genet Evol.

[70] Lim W, Lim F (2021). Corticosteroid management of coronavirus 2019 (COVID-19) in patients with bilateral adrenalectomy. Case Rep Infect Dis.

[71] Isidori AM, Arnaldi G, Boscaro M, Falorni A, Giordano C, Giordano R (2020). COVID-19 infection and glucocorticoids: update from the Italian Society of endocrinology expert opinion on steroid replacement in adrenal insufficiency. J Endocrinol Invest.

[72] Kharroubi AT, Darwish HM (2015). Diabetes mellitus: the epidemic of the century. World J Diabetes.

[73] Seshadri KG, Kirubha MHB (2009). Gliptins: a new class of oral antidiabetic agents. Indian J Pharm Sci.

[74] Patoulias D, Doumas M (2021). Dipeptidyl peptidase-4 inhibitors and COVID-19-related deaths among patients with type 2 diabetes mellitus: a meta-analysis of observational studies. Endocrinol Metab.

[75] Kasina SVSK, Baradhi KM. Dipeptidyl peptidase IV (DPP IV) Inhibitors. 2023; Dpp Iv:1–5. 31194471

[76] Raj VS, Mou H, Smits SL, Dekkers DHW, Müller MA, Dijkman R (2013). Dipeptidyl peptidase 4 is a functional receptor for the emerging human coronavirus-EMC. Nature.

[77] Li Y, Zhang Z, Yang L, Lian X, Xie Y, Li S, et al. The MERS-CoV receptor DPP4 as a candidate binding target of the SARS-CoV-2 spike. iScience. 2020;23. 10.1016/j.isci.2020.101400 PMC7386421 32738607

[78] Vankadari N, Wilce JA (2020). Emerging WuHan (COVID-19) coronavirus: glycan shield and structure prediction of spike glycoprotein and its interaction with human CD26. Emerg Microbes Infect.

[79] RCSB PDB: Homepage. https://www.rcsb.org/. Accessed 18 Jun 2023.

[80] Word JM, Lovell SC, Richardson JS, Richardson DC (1999). Asparagine and glutamine: using hydrogen atom contacts in the choice of side-chain amide orientation. J Mol Biol.

[81] Honorato RV, Koukos PI, Jiménez-García B, Tsaregorodtsev A, Verlato M, Giachetti A (2021). Structural biology in the clouds: the WeNMR-EOSC ecosystem. Front Mol Biosci.

[82] Van Zundert GCP, Rodrigues JPGLM, Trellet M, Schmitz C, Kastritis PL, Karaca E (2016). The HADDOCK2.2 web server: user-friendly integrative modeling of biomolecular complexes. J Mol Biol.

[83] Humphrey W, Dalke A, Schulten KVMD (1996). Visual molecular dynamics. J Mol Graph.

[84] Sebastián-Martín A, Sánchez BG, Mora-Rodríguez JM, Bort A, Díaz-Laviada I (2022). Role of dipeptidyl peptidase-4 (DPP4) on COVID-19 physiopathology. Biomedicines.

[85] Seong JM, Yee J, Gwak HS (2019). Dipeptidyl peptidase-4 inhibitors lower the risk of autoimmune disease in patients with type 2 diabetes mellitus: a nationwide population-based cohort study. Br J Clin Pharmacol.

[86] Nag S, Mandal S, Mukherjee O, Mukherjee S, Kundu R. DPP-4 Inhibitors as a savior for COVID-19 patients with diabetes. 2023. 10.2217/FVL-2022-0112. 10.2217/fvl-2022-0112 PMC10096336 37064327

[87] Ta NN, Li Y, Schuyler CA, Lopes-Virella MF, Huang Y (2010). DPP-4 (CD26) inhibitor alogliptin inhibits TLR4-mediated ERK activation and ERK-dependent MMP-1 expression by U937 histiocytes. Atherosclerosis.

[88] Mozafari N, Azadi S, Mehdi-Alamdarlou S, Ashrafi H, Azadi A (2020). Inflammation: A bridge between diabetes and COVID-19, and possible management with sitagliptin. Med Hypotheses.

[89] Yazbeck R, Jaenisch SE, Abbott CA (2021). Dipeptidyl peptidase 4 inhibitors: Applications in innate immunity?. Biochem Pharmacol.

[90] Birnbaum Y, Tran D, Bajaj M, Ye Y (2019). DPP-4 inhibition by linagliptin prevents cardiac dysfunction and inflammation by targeting the Nlrp3/ASC inflammasome. Basic Res Cardiol.

[91] Sato N, Nakamura Y, Yamadera S, Inagaki M, Kenmotsu S, Saito H (2019). Linagliptin inhibits lipopolysaccharide-induced inflammation concentration-dependently and -independently. J Inflamm Res.

[92] Ran J, Song Y, Zhuang Z, Han L, Zhao S, Cao P (2020). Blood pressure control and adverse outcomes of COVID-19 infection in patients with concomitant hypertension in Wuhan. China Hypertens Res.

