# Supplementary material for: DrugRep-HeSiaGraph: when heterogenous siamese neural network meets knowledge graphs for drug repurposing
Source: BMC Bioinformatics. 2023 Oct 3;24:374. doi: 10.1186/s12859-023-05479-7 (PMC10548718; doi:10.1186/s12859-023-05479-7)
Supplement: Supplementary file 1 — Additional file 1: Table S1. Provide the corresponding evaluation scores of using different combination of drug features, as well as disease features. According to our analysis using Side effect effect as a drug feature and Gene as a disease feature is crucial in facing DR problem. [file 12859_2023_5479_MOESM1_ESM.docx]

DrugRep-HeSiaGraph: When heterogenous siamese neural network meets knowledge graphs for drug repurposing

Zahra Ghorbanali^1^, Fatemeh Zare-Mirakabad^1,^ *, Najmeh Salehi^2^, Mohammad Akbari^1^, Ali Masoudi-Nejad^3^

Corresponding author: [f.zare@aut.ac.ir](mailto:f.zare@aut.ac.ir)

^1^Computational Biology Research Center (CBRC), Department of Mathematics and Computer Science, Amirkabir University of Technology, Tehran, Iran.

^2^School of Biological Science, Institute for Research in Fundamental Sciences (IPM), Tehran, Iran.

^3^Laboratory of Systems Biology and Bioinformatics (LBB), Institute of Biochemistry and Biophysics, University of Tehran, Tehran, Iran.

The Table S1 provide the corresponding evaluation scores of using different combination of drug features, as well as disease features. According to our analysis using Side effect effect as a drug feature and Gene as a disease feature is crucial in facing DR problem.

**Table S 1: Combinations of drug and disease features.**

|  | ACC | AUC-ROC | AUC-PR | BS | MCC | F1 |
| --- | --- | --- | --- | --- | --- | --- |
| **Base Model** | **85.87** | **91.32** | **90.35** | **11.4** | **71.13** | **85.35** |
| **Target** | 73.68 | 81.97 | 80.51 | 17.83 | 47.57 | 71.01 |
| **Domain** | 75.66 | 79.73 | 81.02 | 19.68 | 52.65 | 71.31 |
| **Chemical structure** | 74.34 | 78.87 | 78.43 | 18.38 | 48.66 | 72.72 |
| **ATC** | 75.66 | 84.74 | 84.54 | 16.66 | 51.38 | 73.75 |
| **Side effect** | 82.89 | 89.63 | 90.31 | 13.2 | 65.76 | 82.43 |
| **Target - Domain** | 73.03 | 79.44 | 79.59 | 19.36 | 46.3 | 70.07 |
| **Target - Chemical structure** | 76.32 | 79.98 | 80.65 | 17.95 | 52.63 | 76 |
| **Target - ATC** | 74.34 | 80.93 | 80.67 | 18.16 | 49.14 | 71.11 |
| **Target - Side effect** | 80.92 | 89.18 | 89.39 | 13.62 | 61.8 | 80.27 |
| **Domain- Chemical structure** | 73.03 | 78.52 | 79.66 | 18.91 | 46 | 72.1 |
| **Domain - Side effect** | 83.55 | 89.57 | 90.2 | 13.31 | 67.45 | 82.01 |
| **Domain - ATC** | 71.71 | 78.5 | 78.77 | 19.14 | 43.77 | 68.14 |
| **Chemical structure- Side effect** | 80.26 | 88.33 | 88.94 | 13.86 | 60.52 | 79.16 |
| **Chemical structure - ATC** | 74.34 | 82.59 | 82.04 | 17.04 | 48.63 | 73.1 |
| **Side effect - ATC** | 80.92 | 89.82 | 89.89 | 13.42 | 62.01 | 81.04 |
| **Chemical structure - Target - Side effect** | 80.26 | 88.73 | 89.69 | 13.66 | 60.72 | 78.57 |
| **Chemical structure - Target -ATC** | 75.66 | 82.35 | 82.31 | 17.24 | 51.26 | 74.82 |
| **Chemical structure - Target - Domain** | 71.05 | 77.43 | 79.14 | 19.23 | 42.04 | 69.44 |
| **Chemical structure - Domain - ATC** | 76.97 | 81.93 | 74.37 | 16.9 | 54.03 | 75.17 |
| **Chemical structure - Domain - Side effect** | 84.21 | 90.64 | 87.5 | 11.7 | 68.39 | 83.78 |
| **Chemical structure - Side effect - ATC** | 82.24 | 89.88 | 88.03 | 12.81 | 64.53 | 82.11 |
| **Target - Domain - ATC** | 69.74 | 80.59 | 82.12 | 19.29 | 39.48 | 69.33 |
| **Target - Domain - Side effect** | 84.21 | 91.28 | 89.21 | 11.48 | 68.4 | 83.56 |
| **Target - Side effect - ATC** | 85.53 | 90.28 | 86.83 | 11.81 | 71.07 | 85.33 |
| **Domain - Side effect -ATC** | 84.87 | 90.21 | 85.99 | 11.72 | 69.8 | 84.76 |
| **Without Side effect** | 79.61 | 87.82 | 88.33 | 14.28 | 59.47 | 77.69 |
| **Without ATC** | 84.21 | 91.26 | 89.32 | 11.46 | 68.53 | 84.21 |
| **Without Chemical structure** | 82.24 | 90 | 87.76 | 12.45 | 64.53 | 82.11 |
| **Without Domain** | 84.87 | 91.02 | 88.49 | 11.83 | 69.73 | 84.13 |
| **Without Target** | 82.89 | 90.05 | 86.61 | 12.33 | 65.76 | 82.43 |
| **Only Gene** | 83.55 | 89.72 | 87.41 | 12.53 | 67.07 | 82.99 |
| **Only Semantic type** | 80.64 | 84.92 | 82.43 | 12.95 | 64.33 | 80.14 |
| **Only MeSH class** | 79.98 | 82.65 | 81.73 | 13.56 | 62.21 | 79.11 |
| **Only Type** | 70.01 | 78.22 | 76.21 | 16.78 | 48.86 | 69.78 |
| **Gene – Semantic type** | 84.02 | 89.97 | 88.05 | 12.22 | 68.16 | 83.63 |
| **Gene- MeSH class** | 83.81 | 89.81 | 88.08 | 12.45 | 68.03 | 83.54 |
| **Gene – Type** | 83.45 | 88.72 | 86.81 | 13.33 | 66.52 | 82.1 |
| **Semantic type – MeSH class** | 80.54 | 79.38 | 81.74 | 13.74 | 63.46 | 80.21 |
| **Semantic type – Type** | 73.81 | 81.12 | 80.24 | 14.23 | 62.59 | 79.13 |
| **MeSH class – Type** | 72.19 | 79.92 | 78.13 | 15.12 | 60.37 | 78.06 |
| **without Gene** | 81.58 | 89.25 | 86.66 | 12.89 | 63.17 | 81.33 |
| **Withou Semantic type** | 84.87 | 90.74 | 88.82 | 12.62 | 69.71 | 84.35 |
| **without MeSH class** | 84.21 | 89.91 | 87.5 | 12.48 | 68.44 | 84 |
| **Without Type** | 85.53 | 90.78 | 87.7 | 11.75 | 70.07 | 85.33 |
